# External validation and clinical utility of prognostic prediction models for gestational diabetes mellitus: A prospective cohort study

**DOI:** 10.1111/aogs.13811

**Published:** 2020-02-14

**Authors:** Linda J. E. Meertens, Hubertina C. J. Scheepers, Sander M. J. van Kuijk, Nel Roeleveld, Robert Aardenburg, Ivo M. A. van Dooren, Josje Langenveld, Iris M. Zwaan, Marc E. A. Spaanderman, Marleen M. H. J. van Gelder, Luc J. M. Smits

**Affiliations:** ^1^ Department of Epidemiology, Care and Public Health Research Institute (CAPHRI) Maastricht University Maastricht The Netherlands; ^2^ Department of Obstetrics and Gynecology School for Oncology and Developmental Biology (GROW) Maastricht University Medical Center Maastricht The Netherlands; ^3^ Department of Clinical Epidemiology and Medical Technology Assessment (KEMTA) Maastricht University Medical Center Maastricht The Netherlands; ^4^ Department for Health Evidence Radboud Institute for Health Sciences Radboud University Medical Center Nijmegen The Netherlands; ^5^ Department of Obstetrics and Gynecology Zuyderland Medical Center Heerlen The Netherlands; ^6^ Department of Obstetrics and Gynecology Sint Jans Gasthuis Weert Weert The Netherlands; ^7^ Department of Obstetrics and Gynecology Laurentius Hospital Roermond The Netherlands

**Keywords:** decision curve analysis, external validation, gestational diabetes mellitus, prediction, risk assessment

## Abstract

**Introduction:**

We performed an independent validation study of all published first trimester prediction models, containing non‐invasive predictors, for the risk of gestational diabetes mellitus. Furthermore, the clinical potential of the best performing models was evaluated.

**Material and methods:**

Systemically selected prediction models from the literature were validated in a Dutch prospective cohort using data from Expect Study I and PRIDE Study. The predictive performance of the models was evaluated by discrimination and calibration. Clinical utility was assessed using decision curve analysis. Screening performance measures were calculated at different risk thresholds for the best model and compared with current selective screening strategies.

**Results:**

The validation cohort included 5260 women. Gestational diabetes mellitus was diagnosed in 127 women (2.4%). The discriminative performance of the 12 included models ranged from 68% to 75%. Nearly all models overestimated the risk. After recalibration, agreement between the observed outcomes and predicted probabilities improved for most models.

**Conclusions:**

The best performing prediction models showed acceptable performance measures and may enable more personalized medicine‐based antenatal care for women at risk of developing gestational diabetes mellitus compared with current applied strategies.

**Abbreviations**
AUROC: area under the receiver operating characteristic curve
BMI: body mass index
CI: confidence interval
DM: diabetes mellitus
GDM: gestational diabetes mellitus
LGA: large‐for‐gestational‐age
OGTT: oral glucose tolerance test
PRIDE: PRegnancy and Infant Development
WHO: World Health Organization

**Key message**

Twelve first‐trimester prediction models for the risk of GDM showed moderate predictive performance after external validation. The best performing models may enable more personalized medicine‐based antenatal care for women at risk of developing GDM compared with current applied strategies.

## 1 INTRODUCTION

Gestational diabetes mellitus (GDM) is a common condition during pregnancy. The prevalence increased over the last years and varies considerably between studies (2%‐25%), as it depends on the population studied, the screening method employed and diagnostic criteria used.^1^ GDM is a risk factor for maternal and perinatal complications such as preeclampsia, macrosomia, shoulder dystocia and neonatal hypoglycemia.^2^ Long‐term risks, ie, development of diabetes mellitus (DM) type 2 in both mother and offspring, primarily contribute to the global burden of disease.^3^

Consequences of GDM are often already present at the time of diagnosis (ie, large‐for‐gestational‐age [LGA] infant), as the disorder is mostly asymptomatic.^4^ Therefore, early identification of pregnant women for GDM, usually by an oral glucose tolerance test (OGTT), is essential, as early diagnosis and clinical management improve pregnancy outcomes.^5^ Internationally, however, there is no consensus about whether to screen all women for GDM (universal screening) or only women with prespecified risk factors (selective screening).^6^ Universal screening has a high detection rate but may also lead to an increased burden for women as well as for healthcare resources. Although selective screening reduces the number of women to be screened, a drawback of current risk strategies is that cases are missed at an early stage. Current risk criteria lists are limited by the fact that risk indicators are used independently without taking into account the strength of the different risk factors in relation to GDM.^7^, ^8^ Furthermore, the risk factors are often treated categorically (ie, body mass index >30 kg/m^2^), leading to loss of information that could be obtained using continuous data information.^9^ Prediction models may be more accurate in identifying women at high risk for GDM as multiple risk factors are combined in an algorithm, taking into account the risk‐dependent weight of each risk factor and possible interrelations.^10^ By calculating a probability on a continuous scale, a particular trade‐off between sensitivity and specificity can be chosen. In addition, prognostic prediction models may also constitute a basis for personalized medicine‐based medicine guiding planning of antenatal care and targeting preventive strategies.^11^

A substantial number of prediction models for the risk of GDM have been developed,^12^ but to our knowledge none of these is routinely used in clinical practice. Validation of prediction models in independent populations is a crucial step before implementation in clinical practice.^13^ Only a few studies externally validated models for GDM, and most validated only up to five models.^14^, ^15^, ^16^, ^17^, ^18^ A first comparison of multiple non‐invasive early prediction models for the risk of GDM in an independent cohort was published in 2016.^19^ Most of the prediction models showed acceptable discrimination and calibration.

In this study, we performed a fully independent validation study of all published first trimester prediction models, containing non‐invasive predictors, for the risk of GDM in a Dutch prospective cohort study. In addition, and in contrast to the previous published external validation effort, we evaluated the clinical potential of the best performing models and compared it with the performance of current screening strategies.

## 2 MATERIAL AND METHODS

### 2.1 Selection of prediction models

We performed a systematic search in PubMed to identify prediction models, based on routinely collected parameters and applicable in the first trimester of pregnancy, for the risk of GDM. The search was updated until 13 April 2017. The search strategy and eligibility criteria have been published elsewhere.^20^

### 2.2 Validation cohort

Two population‐based prospective cohorts of pregnant women were used for the validation sample: the Expect Study I and the PRIDE (PRegnancy and Infant DEvelopment) Study. Women with any type of preexisting DM were excluded from the analysis.

#### 2.2.1 Expect Study I

We performed a multicenter prospective cohort study with the primary objective to validate published first‐trimester prediction models for adverse pregnancy outcomes. Six hospitals and 36 midwifery practices in the south‐eastern part of The Netherlands recruited pregnant women less than 16 weeks of gestation and aged 18 years or older between 1 July 2013 and 1 January 2015, with follow up until 31 December 2015. Pregnancies ending in miscarriage, termination at <24 weeks of gestation or for which no outcome data were available, were excluded. Eligible pregnant women were invited to complete two web‐based questionnaires (paper‐based upon request), one at <16 weeks of gestation and one at ≥6 weeks after the due date. Medical records and discharge letters were requested from healthcare providers. A detailed description of the Expect Study I has been published in full elsewhere.^20^

#### 2.2.2 PRIDE Study

The PRIDE Study is an ongoing large, Dutch prospective cohort study among pregnant women. Full details of the study have been published previously.^21^ Pregnant women aged ≥18 years were asked to complete web‐based questionnaires, paper‐based upon request, at baseline (questionnaire 1; 8‐12 weeks of gestation), during gestational weeks 17 (questionnaire 2) and 34 (questionnaire 3), and 2 (questionnaire 4) and 6 (questionnaire 5) months after the due date. Permission was asked to obtain medical records.

Pregnancies enrolled between July 2011 and May 2016 were included in this study. We excluded pregnancies ≥16 weeks of gestation at completion baseline questionnaire, miscarriages, terminations at <24 weeks of gestation and pregnancies with no follow‐up data on outcomes (questionnaire 4 or medical record). If women participated in both studies, the double pregnancy was removed from the PRIDE Study cohort (n = 3).

Medical records were obtained for women who gave permission (~75%) and who had an estimated due date before 1 March 2015.

### 2.3 Predictor variables

The variables in the included prediction models for GDM were extracted from the web‐based questionnaires: pregnancy questionnaire 1 (Expect Study I) and baseline questionnaire (PRIDE Study). In both studies, blood pressure was measured according to routine antenatal care and self‐reported in the questionnaire. In the Expect Study I, most predictor variables were defined according to the original articles. Although the primary goals of the PRIDE Study do not include prediction of pregnancy complications, most predictors were measured similarly. The original articles had different definitions for family history of DM. For comparison and because no distinction was made between the types of DM in the PRIDE Study, we defined two proxy variables for family history of DM: a first‐degree relative with any type of DM and a second‐degree relative with any type of DM. The latter predictor was imputed for PRIDE Study participants, as only family history of first‐degree relatives was assessed. We also redefined the predictor poor obstetric outcome (model Teede et al) as history of antepartum hemorrhage, shoulder dystocia and neonatal death was not administered. A detailed description on predictor definition is provided in Table S1.

### 2.4 Assessment of GDM

Gestational diabetes mellitus was defined as a diagnosis of hyperglycemia during pregnancy in a woman without preexisting DM. According to the World Health Organization (WHO) (1999) guideline, the Dutch national guideline defines hyperglycemia as the presence of either a fasting plasma glucose ≥7.0 mmol/L or 2‐hour plasma glucose ≥7.8 mmol/L following a 75‐g OGTT.^7^ Women at high risk of GDM (prior GDM, body mass index [BMI] >30 kg/m^2^ at first trimester, history of birthweight >95th centile or >4500 g, first‐degree relative with DM, history of unexplained stillbirth, polycystic ovary syndrome, and certain non‐western ethnic groups) are offered an OGTT between 24 and 28 weeks of gestation (selective screening) or if any signs of GDM are present later on in pregnancy (LGA infant or polyhydramnios). A random glucose measurement is recommended in the first trimester to screen for preexisting diabetes.

In both cohorts, the outcome was present in case the postpartum questionnaire or medical record recorded a diagnosis of GDM. For PRIDE Study participants, we also examined questionnaires 2 and 3 for a diagnosis of GDM. In the Expect Study I, we contacted the obstetric care providers in case of discrepancies between the two data sources (n = 29). The postpartum questionnaire was used as reference standard to resolve discrepancies in the PRIDE Study (n = 2).

### 2.5 Statistical analyses

There is no explicit rule for the required sample size for studies externally validating prediction models. Vergouwe et al recommends a minimum of 100 events and 100 non‐events.^22^

Missing data were imputed to prevent biased results.^23^ Stochastic regression imputation with predictive mean matching as the imputation model was used to substitute missing predictor variables in the observed population.

We calculated the individual probabilities of developing GDM for all subjects using the original prediction model algorithms (Table S2). The predictive performance of each model was quantified by measures of discrimination and calibration. We determined discrimination by the area under the receiver operating characteristic curve (AUROC) with 95% confidence interval (CI). Discrimination is the ability of the model to correctly separate women who develop GDM from those who will not. Calibration, the agreement between the predicted probabilities of the model and the observed outcomes, was assessed graphically by calibration plots and by calculation of calibration‐in‐the‐large and the calibration slope. Calibration‐in‐the‐large indicates whether predictions are systematically too high or too low.^10^ The slope measures the average strength of the predictor effects.^10^ The calibration plot should ideally follow the 45° line with an intercept of 0 (calibration‐in‐the‐large) and a slope of 1.^10^ The women were ordered with respect to their predicted probability and subsequently divided into 10 groups of roughly equal size. We recalibrated the prediction models – adjustment intercept and slope – using the linear predictor as the only covariate.^24^ We performed a subgroup analysis among nulliparous women.

For comparability of the models, we used the validation cohort with our inclusion and exclusion criteria. A sensitivity analysis was performed to assess the predictive performance of each model according to their additionally defined eligibility criteria. We also assessed the performance measures in the Expect Study I and the PRIDE Study separately.

The potential clinical utility was evaluated for the best discriminative models by means of decision curve analysis. Decision curve analysis provides insight into the net benefit (net proportion of true positives) of the models over a range of threshold risks as opposed to designating all or no women at high risk of developing GDM.^25^ Finally, we composed a table for the model with the highest net benefit comparing sensitivity, specificity, and positive and negative predictive values for different risk thresholds. Model performance was also compared with that of current selective screening guidelines, the National Institute for Health and Clinical Excellence (NICE) criteria and the Dutch national guideline.^7^, ^8^ Polycystic ovary syndrome, a risk factor according to the Dutch national guideline, was not included, as this predictor was not measured in the Expect Study I.

Statistical analyses were performed with IBM SPSS statistics version 23 (Chicago, IL, USA) and R version 3.2.3, packages rms, pROC, and rmda.

### 2.6 Ethical approval

The Medical Ethical Committee of the Maastricht University Medical Center declared that no ethical approval was necessary for the Expect Study I (MEC 13‐4‐053). The PRIDE Study was approved by the Committee on Research involving Human Subjects region Arnhem‐Nijmegen (CMO 2009/305). Participating women of both studies gave informed consent digitally through the internet.

## 3 RESULTS

### 3.1 Selection of prediction models

The search strategy identified 530 articles. We selected 18 articles that fulfilled the eligibility criteria. We excluded seven papers because the algorithm was not available (n = 3) or the model was already published in one of the included articles (n = 4) (File S1). Reference cross‐checking yielded two additional studies, so 12 articles were included in this validation study.^17^, ^18^, ^26^, ^27^, ^28^, ^29^, ^30^, ^31^, ^32^, ^33^, ^34^, ^35^ The models were published between 1997 and 2017, and were developed in nine different countries. Eight studies used a prospective cohort design, two studies a retrospective cohort design, and two studies were developed in a case‐control study population. Almost all studies (n = 11) used universal screening to detect GDM, but the type of screening strategy differed between the studies. Five studies used a glucose challenge test, four studies a random glucose test, and three studies an OGTT. Gestational diabetes was diagnosed by nearly all studies (n = 9) using a 2‐hour 75‐g OGTT; however, the diagnostic criteria varied between studies. The number of predictors in the published prediction models varied between two and nine. Common predictors were age, BMI, ethnicity, family history of DM, prior macrosomia and prior GDM. A comprehensive overview of the characteristics is available in Table S3.

### 3.2 Validation cohort

The validation cohort included 5260 pregnancies, 2603 pregnancies (2603 women) from the Expect Study I and 2657 pregnancies (2572 women) from the PRIDE Study (Figure S1). GDM was diagnosed in 127 pregnancies (2.4%), 72 pregnancies in the Expect Study I and 55 pregnancies in the PRIDE Study. Twenty‐nine pregnancies complicated by GDM (22.8%) delivered an LGA infant (>90th percentile). The overall prevalence of an LGA infant in the validation cohort was 9.6%. Population characteristics are presented in Table 1. The imputed validation cohort did not materially differ from the observed cohort (with missing data) (Table S4).

**Table 1:** Baseline characteristics of the validation cohort

Characteristics	Missing values, n (%)	Expect Study I (n = 2603)	Missing values, n (%)	PRIDE Study (n = 2657)	Observed validation cohort^a^		
					Overall (n = 5260)	GDM (n = 127)	No GDM (n = 5133)
Age (y)	0 (0.0)	30.2 (3.9)	0 (0.0)	30.6 (3.7)	30.4 (3.8)	31.1 (4.1)	30.4 (3.8)
Ethnicity, n (%)	0 (0.0)		36 (1.4)				
Caucasian		2522 (96.9)		2608 (98.2)	5130 (97.5)	123 (96.9)	5007 (97.5)
Afro‐Caribbean		3 (0.1)		1 (0.0)	4 (0.1)	0 (0.0)	4 (0.1)
Asian		20 (0.8)		6 (0.2)	26 (0.5)	3 (2.4)	23 (.4)
Hispanic		11 (0.4)		2 (0.1)	13 (0.2)	0 (0.0)	13 (0.3)
Mixed		47 (1.8)		4 (0.2)	51 (1.0)	0 (0.0)	51 (1.0)
Tertiary education, n (%)	3 (0.1)	1415 (54.4)	38 (1.4)	2014 (75.8)	3429 (65.2)	65 (51.2)	3364 (65.5)
Height (cm)	3 (0.1)	168.8 (6.4)	17 (0.6)	171.1 (6.3)	170.0 (6.4)	168.8 (6.7)	170.0 (6.4)
Weight (kg)	5 (0.2)	68.9 (13.0)	19 (0.7)	68.6 (11.8)	68.7 (12.4)	78.8 (16.3)	68.5 (12.2)
BMI (kg/m^2^)	5 (0.2)	24.1 (4.3)	25 (0.9)	23.4 (3.8)	23.8 (4.1)	27.7 (6.0)	23.7 (3.9)
Smoking during pregnancy, n (%)	1 (0.0)	156 (6.0)	31 (1.2)	48 (1.8)	204 (3.9)	5 (3.9)	199 (3.9)
History of chronic hypertension, n (%)	0 (0.0)	28 (1.1)	17 (0.6)	2 (0.1)	30 (0.6)	0 (0.0)	30 (0.6)
Family history of diabetes mellitus, n (%)							
First‐degree	1 (0.0)	378 (14.5)	13 (0.5)	292 (11.0)	670 (12.7)	39 (30.7)	631 (12.3)
Second‐degree	1 (0.0)	855 (32.8)	NM	NM	855 (16.3)	31 (24.4)	824 (16.1)
Nulliparous, n (%)	0 (0.0)	1322 (50.8)	0 (0.0)	1442 (54.3)	2764 (52.5)	71 (55.9)	2693 (52.5)
Conception, n (%)	0 (0.0)		15 (0.6)				
Spontaneous		2429 (93.3)		2499 (94.1)	4928 (93.7)	110 (86.6)	4818 (93.9)
Ovulation induction		93 (3.6)		78 (2.9)	171 (3.3)	9 (7.1)	162 (3.2)
IVF/ICSI		81 (3.1)		65 (2.4)	146 (2.8)	6 (4.7)	140 (2.7)
History recurrent miscarriages (≥2), n (%)	0 (0.0)	151 (5.8)	0 (0.0)	124 (4.7)	275 (5.2)	8 (6.3)	267 (5.2)
History of GDM, n (%)	19 (0.7)	14 (0.5)	3 (0.1)	11 (0.4)	25 (0.5)	12 (9.4)	13 (0.3)
History of macrosomia, n (%)							
>90th percentile	52 (2.0)	166 (6.4)	44 (1.7)	218 (8.2)	384 (7.3)	21 (16.5)	363 (7.1)
>4000 g	42 (1.6)	145 (5.6)	61 (2.3)	182 (6.8)	327 (6.2)	15 (11.8)	312 (6.1)
Systolic blood pressure (mm Hg)	260 (10.0)	114 (13)	947 (35.6)	114 (12)	114 (12)	117 (12)	114 (12)
Diastolic blood pressure (mm Hg)	270 (10.4)	68 (9)	953 (35.9)	67 (9)	68 (9)	71 (9)	67 (9)

Abbreviations: GDM, gestational diabetes mellitus; ICSI, intracytoplasmic sperm injection; IVF, in vitro fertilization; NM, not measured.

a Original data (not imputed) presented as mean (SD) for continuous variables or absolute n (%) for categorical variables.

We also evaluated the relatedness between the original cohorts and the validation sample (Table S5). The prevalence of GDM was substantially higher in the original cohort of Phaloprakarn et al (31.2%), Eleftheriades et al (29.9%), Sweeting et al (25.3%) and Tran et al (24.3%). Women in our validation cohort were, in contrast to almost all original cohorts, nearly all of Caucasian origin.

### 3.3 Predictive performance

Table 2 presents the discriminative performance of the included models. Although the AUROC decreased for almost all models compared with the original cohorts, discriminative ability remained satisfactory for all models, with AUROCs ranging from 0.68 to 0.75. The models of Nanda et al and Syngelaki et al yielded the highest discriminative performance (AUROC 0.75, 95% CI 0.70‐0.80 for both models). Application of the models in nulliparous women showed only slight decreases of the AUROCs, except for the model of Gabbay‐Benziv et al (0.05 decline). Sensitivity analyses showed that the models performed similarly in the Expect Study I and the PRIDE Study. Assessment of the discriminative performance of each model using the original population eligibility criteria for selecting the validation sample did not change the AUROC materially (results not shown). The ROC curves of the models in the overall cohort are available in Figure S2A,B.

**Table 2:** Discriminative performance of included prediction models for GDM

Study, first author (year)	AUROC [95% CI]				
	Original publication	Validation cohort (n = 5260)	Validation cohort, nulliparous women (n = 2764)	Expect Study I (n = 2603)	PRIDE Study (n = 2657)
Sweeting (2017)	0.88 [0.85‐0.92]	0.72 [0.67‐0.77]	0.69 [0.62‐0.76]	0.71 [0.65‐0.78]	0.71 [0.63‐0.79]
Syngelaki (2015)	Internal validation: 0.82 [0.82‐0.83]	0.68 [0.62‐0.74]	0.64 [0.56‐0.72]	0.70 [0.62‐0.77]	0.66 [0.56‐0.75]
Eleftheriades (2014)	0.73 [0.65‐0.81]	0.68 [0.63‐0.73]	0.68 [0.60‐0.75]	0.67 [0.60‐0.74]	0.69 [0.61‐0.77]
Gabbay‐Benziv (2014)	0.82 [0.77‐0.87]	0.72 [0.67‐0.77]	0.67 [0.59‐0.75]	0.70 [0.64‐0.77]	0.73 [0.65‐0.81]
Tran (2013)	ADA 0.71 [0.68‐0.75] ADIPS 0.64 [0.62‐0.67] IADPSG 0.65 [0.62‐0.67] WHO 0.63 [0.60‐0.65]	0.70 [0.64‐0.75]	0.69 [0.62‐0.77]	0.68 [0.61‐0.75]	0.71 [0.63‐0.79]
Syngelaki (2011)	NR [CI NR]	0.75 [0.70‐0.80]	0.72 [0.65‐0.80]	0.76 [0.69‐0.82]	0.73 [0.66‐0.81]
Teede (2011)	Internal validation: 0.70 [CI NR]	0.73 [0.68‐0.78]	0.71 [0.63‐0.78]	0.71 [0.64‐0.78]	0.75 [0.67‐0.82]
Nanda (2011)	0.79 [0.76‐0.82]	0.75 [0.70‐0.80]	0.71 [0.64‐0.79]	0.75 [0.68‐0.82]	0.75 [0.67‐0.82]
Van Leeuwen (2010)	0.77 [0.69‐0.85]	0.74 [0.70‐0.79]	0.71 [0.64‐0.78]	0.75 [0.68‐0.81]	0.74 [0.66‐0.81]
Shirazian (2009)	NR [CI NR]	0.71 [0.66‐0.76]	0.71 [0.65‐0.78]	0.70 [0.64‐0.77]	0.71 [0.63‐0.78]
Phaloprakarn (2009)	0.77 [0.75‐0.79] Internal validation: 0.75 [0.73‐0.78]	0.74 [0.69‐0.79]	0.73 [0.66‐0.80]	0.74 [0.67‐0.80]	0.73 [0.66‐0.81]
Naylor (1997)	0.68 [CI NR] Internal validation: NR [CI NR]	0.68 [0.63‐0.73]	0.67 [0.60‐0.74]	0.67 [0.60‐0.73]	0.69 [0.62‐0.77]

Abbreviations: ADA, American Diabetes Association; ADIPS, Australasian Diabetes in Pregnancy Society; AUROC, area under the receiver operating characteristic curve; CI, confidence interval; GDM, gestational diabetes mellitus; IADPSG, International Association of the Diabetes and Pregnancy Study Groups; NR, not reported.

**Figure 1:**
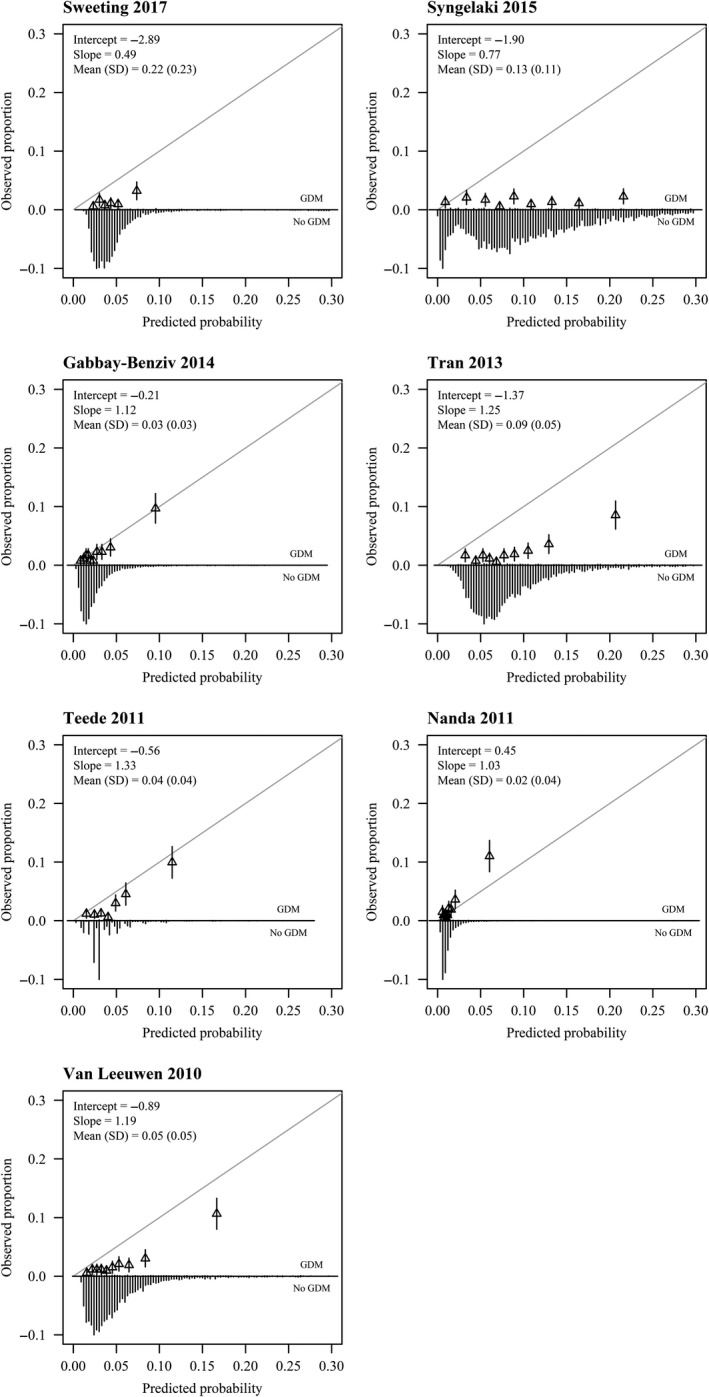
Calibration plots of externally validated first trimester prediction models for gestational diabetes mellitus. The gray line is the reference line with intercept = 0 and slope = 1 (perfect calibration). Triangles correspond to grouped predicted risks with 95% CI (vertical lines)

Calibration plots for the original models that provided a complete prediction algorithm are presented in Figure 1. Models tended to overestimate the risk of GDM (intercept <0), except the model of Nanda et al. The models of Gabbay‐Benziv et al and Nanda et al were the best calibrated. Most models showed better calibration after refitting (Figure S3A,B). The model of Van Leeuwen et al showed the closest fit to the ideal calibration line.

### 3.4 Clinical usefulness

**Figure 2:**
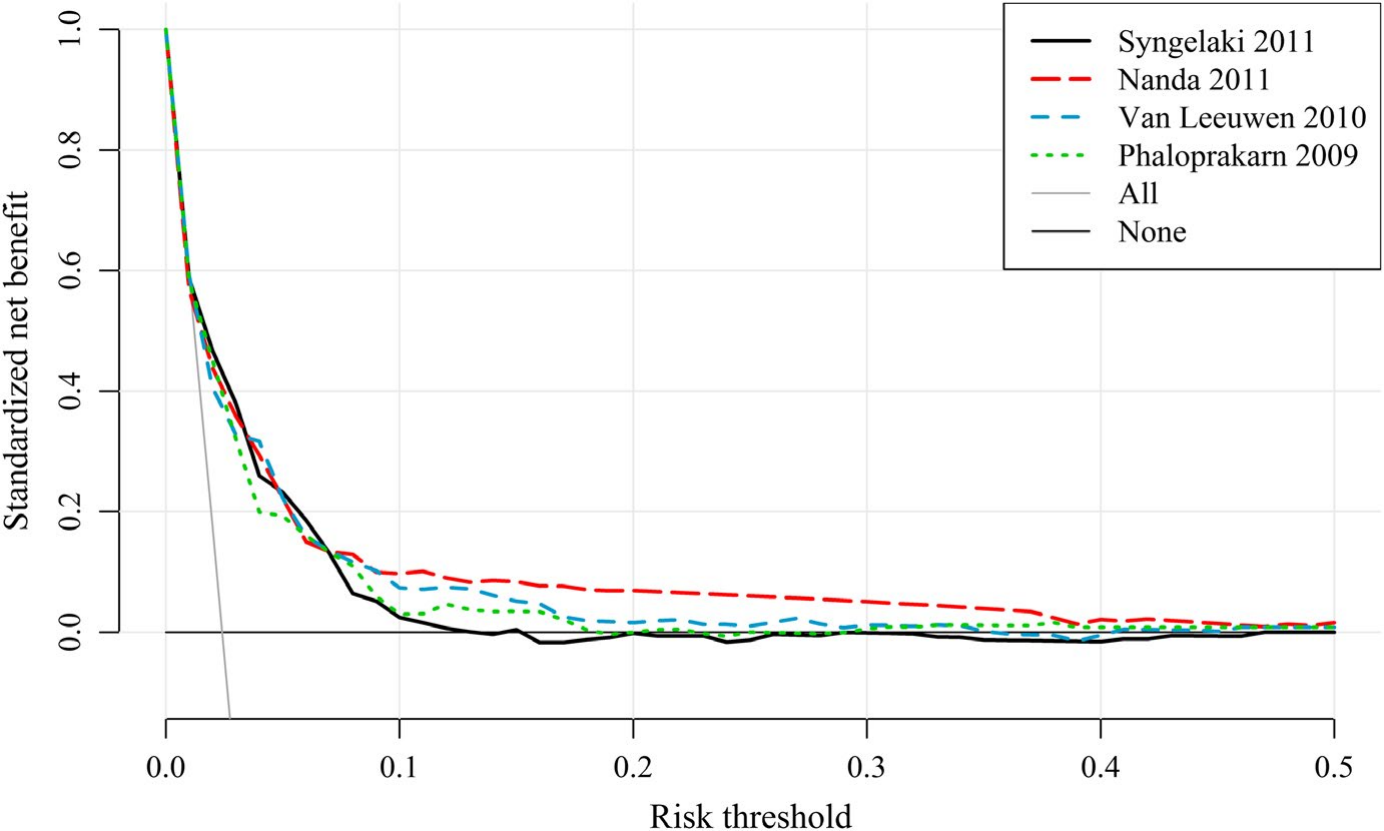
Decision curve analysis of four best performing models for the risk of gestational diabetes mellitus. The solid gray line is the net benefit when considering all women as at high risk and the horizontal black line when considering no women at high risk [Color figure can be viewed at wileyonlinelibrary.com]

Figure 2 shows the decision curve analysis of the four best performing models. These models had a positive net benefit compared with classifying all or no women as at high risk for GDM for a risk threshold ranging between 1% and 55%.

Sensitivity, specificity, and positive and negative predictive values were estimated at different clinically useful risk thresholds for the model of Nanda et al (Table 3). At a low risk threshold (ie, 2%), we observed a high sensitivity and a high negative predictive value, suggesting a strong ability to rule out GDM in women who are indicated as low risk. At this high sensitivity, however, a lot of women will be unnecessarily indicated as having a high risk (high false‐positive rate). A risk threshold above 5% leads to a drastically low sensitivity, so a large proportion of women that will develop GDM would be incorrectly classified as having a low risk.

**Table 3:** Sensitivities, specificities and predictive values at different risk thresholds for the model of Nanda et al

Risk threshold,^a^ %	High risk, %	Sensitivity, %	Specificity, %	PPV, %	NPV, %
1	90.5	93.7	9.6	2.5	98.4
2	35.6	72.4	65.3	4.9	99.0
3	16.3	55.1	84.6	8.1	98.7
4	9.1	43.3	91.7	11.4	98.5
5	5.4	32.3	95.3	14.5	98.3
10	1.1	13.4	99.2	28.8	97.9
20	0.5	9.4	99.7	48.0	97.8
40	0.4	7.9	99.8	47.6	97.8

Abbreviations: NPV, negative predictive value; PPV, positive predictive value.

a Predicted risk at or above this level was considered to be high risk.

We compared the model of Nanda et al with current selective screening guidelines. The NICE criteria classified 21% of the women as at high risk in the validation cohort with a sensitivity of 62% and a specificity of 80%. When applying the Dutch national guideline criteria to the validation cohort, 23% of the women were considered to be at high risk for developing GDM with a sensitivity of 65% and a specificity of 78%. The model of Nanda et al had similar specificities at the same sensitivities and vice versa, but higher values can be achieved when selecting another risk threshold (Table 3).

## 4 DISCUSSION

We validated 12 prediction models for the risk of GDM in a Dutch prospective cohort. All models had a moderate discriminative performance with an AUROC around 0.70. The best discriminating models were those of Syngelaki et al and Nanda et al (AUROC 0.75). Nearly all models overestimated the risk of developing GDM in our cohort. Recalibration led to better agreement between actual risks and predicted probabilities for most models.

External validation is important, as prediction models generally perform too optimistically in the development sample.^24^ The discriminative performance decreased for all models except those of Tran et al, Teede et al and Naylor et al. A history of GDM is strongly associated with the risk of GDM; nevertheless, the discriminative performance of most models was not lower in the subgroup analysis including only nulliparous women.

Previous external validation studies that validated only a few models yielded similar results for the models of Nanda et al, Teede et al, Van Leeuwen et al and Naylor et al.^14^, ^16^, ^17^, ^18^ The only published study that also performed a comprehensive external validation of multiple prediction models showed slightly higher discriminative performances compared with our results.^19^ They concluded that the model of Teede et al and Van Leeuwen et al had the best overall performance. We validated three additional models based on maternal characteristics and evaluated the clinical potential of the best performing models compared with current screening strategies as suggested in their discussion.

Multiple external validation of a prediction model adds to the robustness of model performance.^24^ Two of our best performing models, Nanda et al and Van Leeuwen et al, showed similar performances in several independent populations. The other comprehensive external validation study was performed in a Dutch population as well, but from another geographic area. This strengthens the generalizability of the models to the general Dutch population and even to antenatal populations in other high‐income countries.

A prognostic prediction model identifies women at risk for developing GDM compared with diagnostic models that detect diabetes. By providing objective individual estimates, healthcare providers and women can be guided regarding decision making towards personalized follow‐up management.

When screening for GDM using an OGTT, a prediction model may be more beneficial than current selection strategies. The proportion of women with identified GDM increases with the number of women offered an OGTT, irrespective of the screening strategy used.^36^ Universal screening leads to 100% detection, but the majority of women have to undergo an OGTT that may place an unnecessarily burden on individual women and healthcare resources. Current selective screening strategies are based on a list of risk factors and have a fixed sensitivity (± 65%) and specificity (± 80%).^7^, ^8^ Although the best performing models do not provide more benefit at certain risk thresholds compared with current available screening strategies, an advantage is that a preferred trade‐off between sensitivity and specificity can be selected. For example, if a sensitivity of 80% is chosen, 50% of the women must undergo an OGTT. Determination of an acceptable risk threshold is a challenging aspect of clinical usefulness. The choice for a specific risk threshold depends on several factors, such as consequences of the outcome, the effect of treatment of GDM, burden of OGTT and related costs. Short‐ and long‐term consequences of GDM are well known and treatment is proven to be effective.^3^, ^5^ However, robust evidence is lacking on reduction of more serious maternal and perinatal complications as well as on the long‐term benefit of treatment, such as reduced incidence of type 2 DM.^5^

Moreover, a prognostic prediction model provides opportunities for allocating preventive measures. Maternal pre‐pregnancy BMI and gestational weight gain are associated with the risk of developing GDM.^37^ Despite emerging promising studies of preventive interventions, such as lifestyle interventions, no hard evidence is available yet.^11^ The limited available studies have methodological shortcomings such as heterogeneity of the interventions and small sample size.^6^, ^11^ Nevertheless, awareness and interventions to lead to a healthy lifestyle are essential means in the prevention of GDM in our opinion, which can be advised without causing harm.

In the end, only an impact study can determine whether the model contributes to improved personalized care, since this depends on several other aspects, such as participant and care givers’ behavior and management, risk counseling and related costs.^38^

The main strengths of our study are the large sample size, sufficient number of cases and the multicenter prospective cohort design. A cohort study represents the most powerful design for external validation, but selection may bias the generalizability of the results.^39^ The Expect Study I and PRIDE Study have relatively low response rates (~30%) and women with a high educational level (national prevalence 2014; 48%) and of Caucasian origin were overrepresented.^20^, ^40^, ^41^ A recent Danish birth cohort study showed that this may not affect exposure‐outcome associations substantially. Dropouts and missing data during follow up are more harmful and should be avoided as much as possible rather than prioritizing representativeness.^42^ High data quality and low quantity of missing data were achieved by the use of web‐based questionnaires. Nevertheless, blood pressure measurements had a substantial amount of missing values as a result of self‐report.^43^ The predictor blood pressure was, however, only necessary for one included prediction model. Missing data were imputed to prevent biased results. Next, we had to generate proxy variables for family history of DM. Although a positive family history of second‐degree relatives was imputed completely for the PRIDE Study cohort, no differences in the predictive performance of the models containing this predictor were observed between the Expect Study I and PRIDE Study.

Another limitation to be mentioned is that the OGTT was only performed as a screening tool in women at high risk for GDM according to the Dutch national guideline.^7^ Nevertheless, diagnosis of GDM was based on review of medical records and the postpartum questionnaire, which allowed us to detect all diagnosed cases of GDM, including late diagnosis of GDM. In our study, 65% of the women with a diagnosis of GDM fulfilled the Dutch criteria of screening, indicating that 35% of our cases were most likely detected outside of selective screening (ie, glucose measurement after sonographic diagnosis of fetal macrosomia or polyhydramnios). Still, cases of GDM may have been missed in asymptomatic women. False‐negatives can lead to an underestimation of the c‐statistic.^44^ Nationwide data on the prevalence of GDM in the Netherlands are scarce, but estimated prevalence varies between 2% and 5%. A study of Van Leeuwen et al, in which universal screening with the same diagnostic criteria was performed in a fairly comparable Dutch pregnant population, showed a similar prevalence of GDM.^32^ We recognize that this prevalence is low compared with other countries. A meta‐analysis reported an overall prevalence in Europe of 5.4% (3.8%‐7.8%), with lowest prevalence in Northern Europe.^45^ Prevalence rates are affected by different screening and diagnostic criteria used as well as population characteristics.^46^ Internationally there is no consensus regarding the optimal cut‐off points for diagnosing GDM. Prevalence rates are substantially higher when using lower glucose levels as recommended by the International Association of the Diabetes and Pregnancy Study Groups (IADPSG).^47^ Tran et al calculated the discriminative performance of the model for different diagnostic criteria and showed no substantial difference between the IADPSG and WHO 1999 criteria.^28^ In the end, a head‐to‐head comparison, as performed in this study, allows for a fair comparison of the performance of prediction models in a particular population with specific screening and diagnostic criteria and is necessary before a model can be implemented in clinical practice.

## 5 CONCLUSION

The best performing prediction models showed acceptable performance measures and may enable more personalized medicine‐based antenatal care for women at risk of developing GDM compared with current applied strategies. A next step is to investigate the impact of implementation of the best model with risk‐dependent care in clinical practice.

## CONFLICT OF INTEREST

None.
