# Supplementary figures and images for: External validation and clinical utility of prognostic prediction models for gestational diabetes mellitus: A prospective cohort study
Source: Acta Obstet Gynecol Scand. 2020 Feb 14;99(7):891–900. doi: 10.1111/aogs.13811 (PMC7317858; doi:10.1111/aogs.13811)

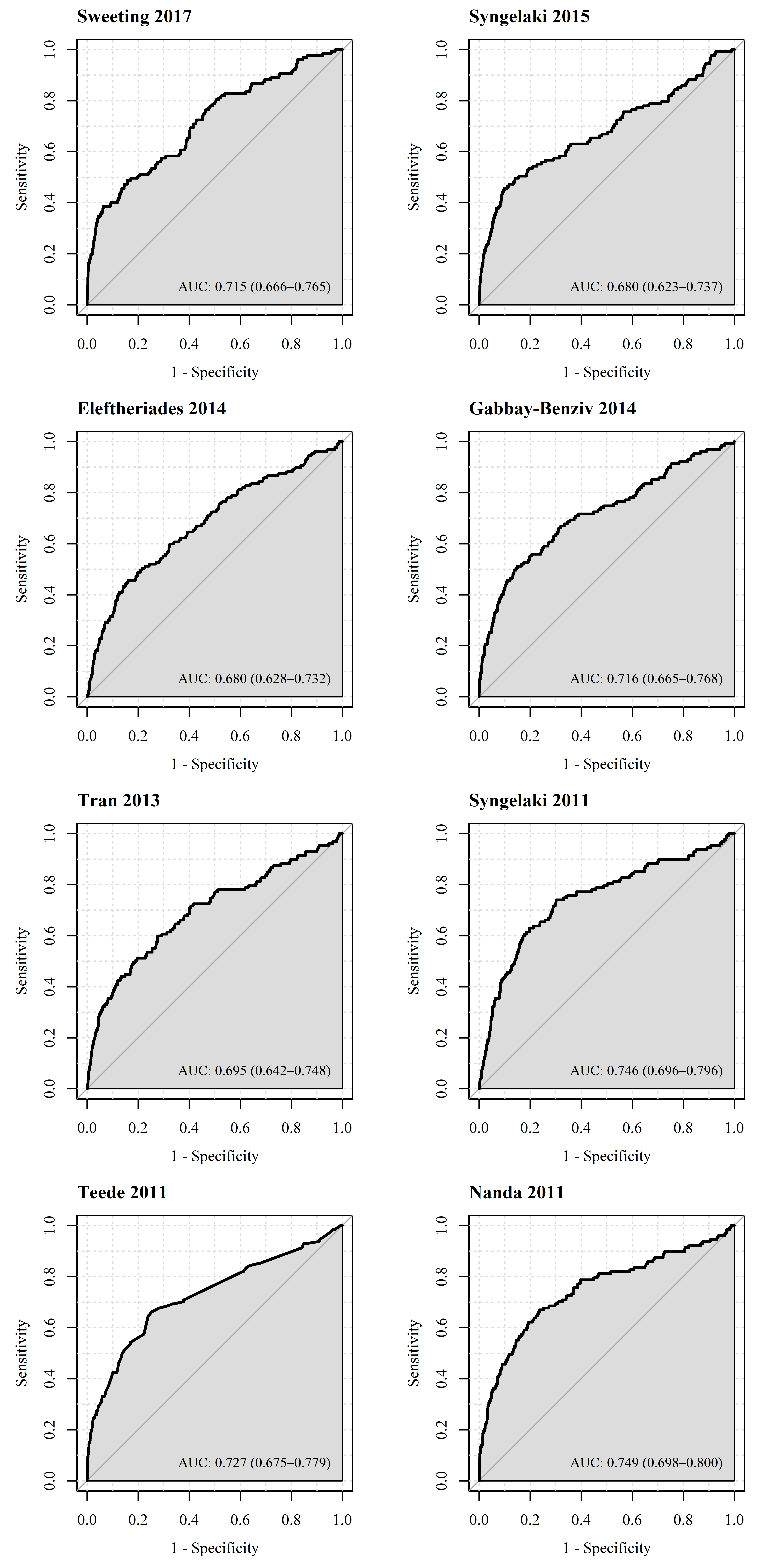

Supplement: Supplementary file 2 [file AOGS-99-891-s002.tif]

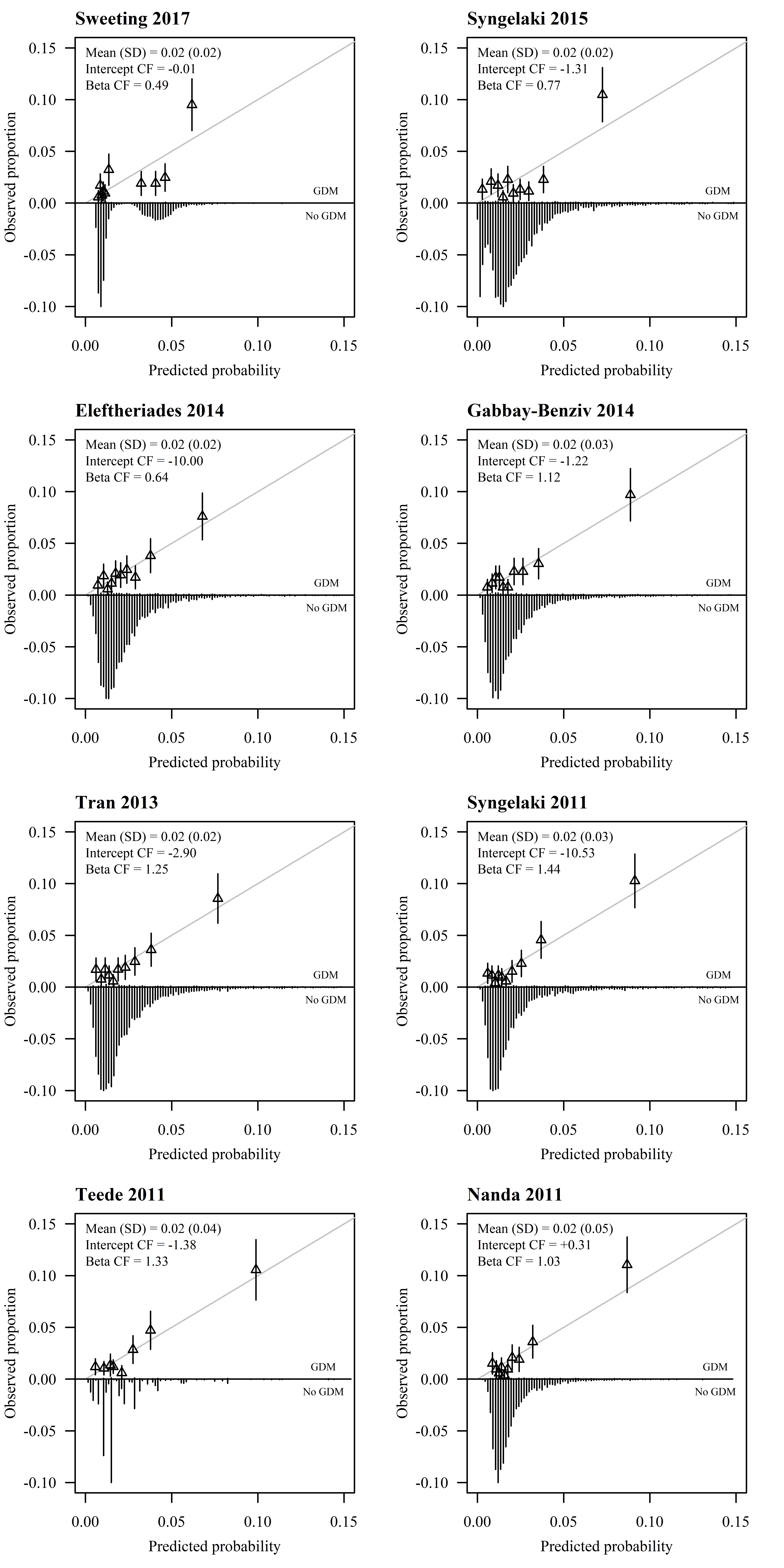

Supplement: Supplementary file 3 [file AOGS-99-891-s003.tif]
