# Supplementary material for: External validation and clinical utility of prognostic prediction models for gestational diabetes mellitus: A prospective cohort study
Source: Acta Obstet Gynecol Scand. 2020 Feb 14;99(7):891–900. doi: 10.1111/aogs.13811 (PMC7317858; doi:10.1111/aogs.13811)
Supplement: Supplementary file 4 [file AOGS-99-891-s004.docx]

Table S1. Definition and assessment of predictors

| **Predictor** | **Definition (D)/measurement (M) original studies** | **Definition/measurement validation cohort**  **(Expect Study I)** | **Definition/measurement validation cohort**  **(PRIDE Study)** |
| --- | --- | --- | --- |
| Age (years) | Sweeting 2017  D: Continuous in years  M: Recorded at the time of first trimester screening between 11^+0^-13^+6^ weeks of gestation.  Syngelaki 2015  D: Continuous in years – 35.  M: Self-reported questionnaire 11^+0^-13^+6^ weeks of gestation.  Eleftheriades 2014  D: Continuous in years.  M: Recorded at the time of the combined screening for aneuploidies by ultrasound scan and maternal serum biochemistry.  Gabbay-Benziv 2014  D: Continuous in years.  M: Age at enrolment.  Tran 2013  D: Continuous in years.  M: 10-min intervieuw at the time of OGTT testing conducted by one of three trained research midwives.  Syngelaki 2011, Nanda 2011  D: Continuous in years.  M: Self-reported questionnaire 11^+0^-13^+6^ weeks of gestation.  Teede 2011  D: Categories in years: <25, 25-29, 30-34, 35-39, and ≥40.  M: The Birthing Outcomes System, an electronic database at Monash Medical Centre.  Shirazian 2009  D: Categories in years: ≤24, 25-29, and >30.  M: Pregnant women responded to a structured questionnaire at the first prenatal visit.  Phaloprakarn 2009  D: Continuous in years.  M: Maternal age was assigned in the whole number of years (rounding off months and days) at the time of last menstrual period.  Naylor 1997  D: Categories in years: ≤30, 31-34, and ≥35.  M: Not reported. | Self-reported pregnancy questionnaire 1, date of birth. Obstetric records were checked for discrepancies. Age calculated from date of birth, continuous in years. Categorical variables generated according to definition original prediction model. | Calculated from date of birth (informed consent), continuous in years. Categorical variables generated according to definition original prediction model. |
| Ethnicity | Sweeting 2017  D: Ethnicity divided into Caucasian, East Asian, South Asian and other (Middle Eastern, African, or combination of south-east Asian and Caucasian).  M: Recorded at the time of first trimester screening between 11^+0^-13^+6^ weeks of gestation.  Syngelaki 2015, Syngelaki 2011, Nanda 2011  D: Ethnic origin divided into Caucasian, Afro-Caribbean, Indian or Pakistani or Bangladeshi (South Asian), Chinese or Japanese (East Asian) and mixed.  M: Self-reported questionnaire 11^+0^-13^+6^ weeks’ gestation.  Gabbay-Benziv 2014  D: Maternal demographics (white, black, Asian, Hispanic, other).  M: At the first visit after enrolment.  Teede 2011  D: Ethnicity divided into Anglo-Australian, Polynesian, Mainland South-East Asian, Maritime South-East Asian, Chinese Asian, Southern Asian, African, and Other (Middle Eastern, Central Asian, European and American origins).  M: The Birthing Outcomes System, an electronic database at Monash Medical Centre.  Van Leeuwen 2010  D: Caucasian and Non-Caucasian ethnicity.  M: Self-reported at intake.  Naylor 1997  D: Ethnicity was categorized as white, black, Asian or other. Other included women of Hispanic, South Asian (e.g., from India, Pakistan, Sri Lanka, and Bangladesh), and Middle Eastern origins.  M: Interview by study nurses. | Self-reported pregnancy questionnaire 1, ethnicity was divided into 10 subgroups: Dutch, Turkish/Kurdish, Moroccan (Moroccan, Algerian, North African), African (African, Surinamese/Antillean of Negroid origin), Hindustani (Hindustani, Pakistani, Indian, Surinamese / Antillean of Hindu origin), Middle East (Iran, Iraqi, Afghan), Asian (Chinese, Japanese, Indonesian, Albanian, Vietnamese), Other Western (European, North American, Australian), Other Non-Western (South and Central American), and mixed.  Ethnicity was recoded to Caucasian, Asian, Afro-Caribbean, Hispanic, and mixed (combination of other categories). Subdivision of Asian ethnicity was based on country of birth biological parents.  Syngelaki 2011: we added women with an Asian ethnicity other than South Asian or East Asian to the category mixed. Hispanics were categorized as Caucasians.  Gabbay-Benziv 2014 and Naylor 1997: women with mixed ethnicity were categorized as other. | Self-reported pregnancy questionnaire 1, ethnicity was divided into 15 subgroups: Dutch, European/North American, Surinamese Creole, Surinamese other, Antillean Creole, Antillean other, Turkish, Kurdish, Moroccan, Berber, Indonesian, Asian, African, South American, and other.  Ethnicity was recoded to Caucasian, Asian, Afro-Caribbean, Hispanic, and mixed (combination of other categories). Subdivision of Asian ethnicity was based on country of birth biological parents.  Syngelaki 2011: we added women with an Asian ethnicity other than South Asian or East Asian to the category mixed. Hispanics were categorized as Caucasians.  Gabbay-Benziv 2014 and Naylor 1997: women with mixed ethnicity were categorized as other. |
| Weight (kg) | Syngelaki 2015  D: Continuous in kg – 69.  M: Weight measured at routine assessment at 11^+0^-13^+6^ weeks of gestation.  Eleftheriades 2014  D: Continuous in kg.  M: Recorded at the time of the combined screening for aneuploidies by ultrasound scan and maternal serum biochemistry. | Self-reported pregnancy questionnaire 1, pre-pregnancy weight in kilograms. | Self-reported pregnancy questionnaire 1, pre-pregnancy weight in kg. The weight at completion of baseline questionnaire was taken in the case of a missing value (n=188). |
| Height (cm) | Syngelaki 2015  D: Continuous in cm – 164.  M: Height measured at routine assessment at 11^+0^-13^+6^ weeks of gestation. | Self-reported pregnancy questionnaire 1, height in cm. | Self-reported pregnancy questionnaire 1, height in cm. |
| BMI (kg/m^2^) | Sweeting 2017  D: Continuous in kg/m^2^.  M: Height and weight was measured by a midwife at the time of first trimester screening between 11^+0^-13^+6^ weeks of gestation.  Gabbay-Benziv 2014  D: Continuous in kg/m^2^.  M: Prepregnancy maternal weight and height obtained at the first visit after enrolment. BMI was calculated as weight (kg)/height (m)^2^.  Tran 2013  D: Continuous in kg/m^2^ at booking.  M: Weight and height were determined from the antenatal record and measured again at the time of OGTT. Body weight was measured in light clothing without shoes, and height was determined without shoes on a portable stadiometer with a mandible plane parallel to the floor.  Syngelaki 2011, Nanda 2011  D: Continuous in kg/m^2^.  M: Height and weight measured at routine assessment at 11^+0^-13^+6^ weeks’ gestation.  Teede 2011  D: Categories in kg/m^2^: <20.0, 20.0-24.9, 25.0-26.9, 27.0-29.9, 30.0-34.9, and ≥35.0.  M: Weight and height measured in early pregnancy at the initial hospital visit by midwifery staff.  Van Leeuwen 2010  D: Continuous in kg/m^2^.  M: Height and weight were obtained at intake. BMI before pregnancy was calculated as weight (kg)/[height (m)]^2^.  Shirazian 2009  D: Categories in kg/m^2^: ≤24.9, 25.0-29.9, and ≥30.0.  M: Pregnant women responded to a structured questionnaire at the first prenatal visit. Each woman’s pre-pregnancy BMI was calculated from last height and most recent weight before conception.  Phaloprakarn 2009  D: Continuous in kg/m^2^.  M: first-visit BMI was calculated from weight and height, which were measured using the same equipment at the antenatal clinic for all women.  Naylor 1997  D: Categories in years: ≤22.0, 22.1-25.0, and ≥25.1.  M: Each woman’s BMI immediately before pregnancy was calculated from her self-reported height and most recent weight before conception. BMI was calculated as the weight in kilograms divided by the square of the height in meters. | Self-reported pregnancy questionnaire 1, height in centimetres and pre-pregnancy weight in kilograms. BMI was calculated as weight (kilograms) divided by the squared height (meters). BMI continuous in kg/m^2^, transformed into categorical variable according to definition original prediction model. | Self-reported pregnancy questionnaire 1, height in centimetres and pre-pregnancy weight in kilograms. BMI was calculated as weight (kilograms) divided by the squared height (meters). BMI continuous in kg/m^2^, transformed into categorical variable according to definition original prediction model. |
| Smoking | Syngelaki 2011  D: Cigarette smoking during pregnancy.  M: Self-reported questionnaire 11^+0^-13^+6^ weeks’ gestation. | Self-reported pregnancy questionnaire 1, cigarette smoking status (non-smoker, stopped during pregnancy, current smokers).  Cigarette smoking was defined as current smoker at completion pregnancy questionnaire 1. | Self-reported pregnancy questionnaire 1, cigarette smoking status (non-smoker, stopped during pregnancy, current smokers).  Cigarette smoking was defined as current smoker at completion pregnancy questionnaire 1. |
| History of chronic hypertension | Syngelaki 2011  D: History of chronic hypertension.  M: Self-reported questionnaire 11^+0^-13^+6^ weeks’ gestation. | Self-reported pregnancy questionnaire 1, chronic hypertension. | Self-reported pregnancy questionnaire 1, chronic hypertension. |
| Family history of diabetes mellitus | Sweeting 2017  D: Family history of diabetes (defined as type 2 diabetes in a first or second degree relative and/or sibling with GDM).  M: Self-reported questionnaire 11^+0^-13^+6^ weeks of gestation  Syngelaki 2015  D: Family history of diabetes mellitus (first or second degree relative with diabetes mellitus type 1 or 2).  M: Self-reported questionnaire 11^+0^-13^+6^ weeks of gestation.  Teede 2011  D: First-degree family history of diabetes type 2.  M: The Birthing Outcomes System, an electronic database at Monash Medical Centre.  Van Leeuwen 2010  D: Family history of diabetes mellitus (defined as a first- or second-degree relative with diabetes mellitus type I or II).  M: Obtained at intake.  Shirazian 2009  D: Family history of diabetes type 2 in first degree relatives.  M: Pregnant women responded to a structured questionnaire at the first prenatal visit.  Phaloprakarn 2009  D: History of diabetes type 2 in any first-degree relatives.  M: Gestational diabetes mellitus screening. | Self-reported pregnancy questionnaire 1, diabetes mellitus woman’s biological mother, biological father, biological grandparents, biological brother or biological sister. We separately asked to type of diabetes (1 or 2).  First degree relative was defined as a parent or sibling with diabetes mellitus and second degree as a grandparent with diabetes mellitus.  Sweeting 2017: Family history of diabetes mellitus was defined as first or second degree relative with any type of diabetes mellitus.  Teede 2011, Shirazian 2009, Phaloprakarn 2009: Any type of diabetes mellitus in first degree relatives was considered as positive for family history of diabetes mellitus. | Self-reported pregnancy questionnaire 1, diabetes mellitus woman’s biological mother, biological father, biological brother or biological sister. There was no distinction made between the type of diabetes. First degree relative was defined as a parent or sibling with diabetes mellitus.  The variable family history of diabetes mellitus type 1 or 2 in second degree relatives was imputed based on the Expect Study I data.  Sweeting 2017: Family history of diabetes mellitus was defined as first or second degree relative with any type of diabetes mellitus.  Teede 2011, Shirazian 2009, Phaloprakarn 2009: Any type of diabetes mellitus in first degree relatives was considered as positive for family history of diabetes mellitus. |
| Parity | Sweeting 2017  D: Multiparous or nulliparous if a woman had no previous pregnancies at or beyond 24 weeks of gestation.  M: Recorded at the time of first trimester screening between 11^+0^-13^+6^ weeks of gestation.  Syngelaki 2015, Nanda 2011  D: Parous or nulliparous with no pregnancies at or beyond 24 weeks of gestation.  M: Self-reported questionnaire 11^+0^-13^+6^ weeks’ gestation.  Syngelaki 2011  D: Obstetric history including the outcome of each pregnancy.  M: Self-reported questionnaire 11^+0^-13^+6^ weeks of gestation.  Van Leeuwen 2010  D: Parity.  M: Obtained at intake. | Self-reported pregnancy questionnaire 1 and checked for discrepancies by obstetric record.  The Dutch definition was used: every delivery ≥16 weeks of gestation. | Self-reported pregnancy questionnaire 1. The Dutch definition was used: every delivery ≥16 weeks of gestation. |
| Method of conception | Syngelaki 2015, Syngelaki 2011  D: Method of conception (spontaneous or assisted conception requiring the use of ovulation drugs).  M: Self-reported questionnaire 11^+0^-13^+6^ weeks of gestation. | Self-reported pregnancy questionnaire 1, method of conception (spontaneous, use of ovulation drugs or in vitro fertilization). | Self-reported pregnancy questionnaire 1, method of conception (spontaneous, use of ovulation drugs or in vitro fertilization). |
| History of ≥2 abortions | Phaloprakarn 2009  D: History of ≥2 spontaneous abortions.  M: Gestational diabetes mellitus screening. | Self-reported pregnancy questionnaire 1, history of miscarriages and weeks of gestation, and checked for discrepancies by obstetric record. | Self-reported pregnancy questionnaire 1, history of miscarriages and weeks of gestation. |
| History of gestational diabetes mellitus (GDM) | Sweeting 2017  D: Previous history of GDM.  M: Self-reported questionnaire 11^+0^-13^+6^ weeks of gestation.  Syngelaki 2015, Nanda 2011  D: Any of the previous pregnancies were complicated by GDM.  M: Self-reported questionnaire 11^+0^-13^+6^ weeks of gestation.  Gabbay-Benziv 2014  D: Prior GDM (obstetric history).  M: At the first visit after enrolment.  Teede 2011  D: Past history of GDM.  M: The Birthing Outcomes System, an electronic database at Monash Medical Centre.  Van Leeuwen 2010  D: History of GDM.  M: Obtained at intake. | Self-reported pregnancy questionnaire 1, gestational diabetes in previous pregnancy. | Self-reported pregnancy questionnaire 1, gestational diabetes in previous pregnancy. |
| History of large-for-gestational-age (LGA) infancy | Syngelaki 2011  D: Obstetric history including the outcome of each pregnancy. LGA was defined as those with birth weight above the 95^th^ percentile for gestation.  M: Self-reported questionnaire 11^+0^-13^+6^ weeks’ gestation.  Nanda 2011  D: Obstetric history including the outcome of each pregnancy. LGA was defined as those with birth weight above the 90^th^ percentile for gestational age.  M: Self-reported questionnaire 11^+0^-13^+6^ weeks’ gestation. | Self-reported pregnancy questionnaire 1, birth weight of each previous pregnancy. The percentile was calculated corrected for gestational age, ethnicity, gender, and parity^2^. Percentile transformed into categorical variable according to definition original prediction model. | Self-reported pregnancy questionnaire 1, birth weight of each previous pregnancy. The percentile was calculated corrected for gestational age, ethnicity, gender, and parity.^2^ Percentile transformed into categorical variable according to definition original prediction model. |
| History of macrosomic infant | Phaloprakarn 2009  D: Prior delivery of a macrosomic infant (≥4000 g).  M: Gestational diabetes mellitus screening. | Self-reported pregnancy questionnaire 1, birth weight of each previous pregnancy. Birth weight continuous in grams, transformed into categorical variable according to definition original prediction model. | Self-reported pregnancy questionnaire 1, birth weight of each previous pregnancy. Birth weight continuous in grams, transformed into categorical variable according to definition original prediction model. |
| Birth weight z-score previous pregnancy | Syngelaki 2015  D: Birth weight z-score of the last pregnancy with delivery at or beyond 24 weeks, continuous. The z-score is the difference between the observed and expected birth weight for gestational age.^1^  M: Self-reported questionnaire 11^+0^-13^+6^ weeks’ gestation. | Self-reported pregnancy questionnaire 1, birth weight of last pregnancy. The z-score was calculated as birth weight minus birth weight mean divided by the standard deviation (SD), using birth weight means and SDs from the Dutch population standards corrected for gestational age, ethnicity, gender, and parity.^2^ | Self-reported pregnancy questionnaire 1, birth weight of last pregnancy. The z-score was calculated as birth weight minus birth weight mean divided by the standard deviation (SD), using birth weight means and SDs from the Dutch population standards corrected for gestational age, ethnicity, gender, and parity.^2^ |
| History of poor obstetric outcome | Teede 2011  D: Poor obstetric outcome was classiﬁed as any adverse outcome considered potentially related to hyperglycaemia – fetal death, neonatal death, fetal abnormalities, intrauterine growth restriction, shoulder dystocia, preterm birth, pre-eclampsia, antepartum haemorrhage and multiple miscarriages (≥3).  M: The Birthing Outcomes System, an electronic database at Monash Medical Centre. | Self-reported pregnancy questionnaire 1, history of stillbirth, neonatal mortality within first 24 hours of life, history of birth weight below the 10^th^ percentile, prior delivery before 37 weeks of gestation, pre-eclampsia in previous pregnancy, and history of miscarriages and weeks of gestation. We did not specifically asked history of fetal abnormalities, antepartum haemorrhage and shoulder dystocia. Fetal abnormality was defined as termination of a previous pregnancy because of fetal abnormality. We also checked if fetal anomaly in previous pregnancy was mentioned in the obstetric record. History of miscarriages was also checked for discrepancies by obstetric record.  Teede 2011: Poor obstetric outcome was redefined as positive in case of history of fetal death, fetal abnormalities, intrauterine growth restriction (<10^th^ percentile), preterm birth before 37 weeks of gestation, pre-eclampsia, or multiple miscarriages (≥3). | Self-reported pregnancy questionnaire 1, history of stillbirth, fetal abnormalities (live birth and stillborn children, and miscarriage or termination because of fetal abnormality), history of birth weight below the 10^th^ percentile, prior delivery before 37 weeks of gestation, pre-eclampsia in previous pregnancy, and history of miscarriages and weeks of gestation.  There was not asked to history of neonatal mortality, antepartum haemorrhage, and shoulder dystocia.  Teede 2011: Poor obstetric outcome was redefined as positive in case of history of fetal death, fetal abnormalities, intrauterine growth restriction (<10^th^ percentile), preterm birth before 37 weeks of gestation, pre-eclampsia, or multiple miscarriages (≥3). |
| Systolic blood pressure | Gabbay-Benziv 2014  D: Continuous in mmHg.  M: Blood pressure was obtained at the first visit after enrolment. | Blood pressure (systolic and diastolic, mmHg) was measured by the gynaecologist or midwife following standard procedure before 16 weeks of gestation. The results of these measurements (with date) were provided to the eligible women in order to self-report in pregnancy questionnaire 1. | Self-reported pregnancy questionnaire 1, blood pressure measurement (systolic and diastolic, mmHg) at last antenatal visit (date). |

**References**

1. Poon LC, Volpe N, Muto B, Syngelaki A, Nicolaides KH. Birthweight with gestation and maternal characteristics in live births and stillbirths. Fetal Diagn Ther. 2012;32:156-65.

2. Visser GH, Eilers PH, Elferink-Stinkens PM, Merkus HM, Wit JM. New Dutch reference curves for birthweight by gestational age. Early Hum Dev. 2009;85:737-44.
