# Supplementary material for: External validation and clinical utility of prognostic prediction models for gestational diabetes mellitus: A prospective cohort study
Source: Acta Obstet Gynecol Scand. 2020 Feb 14;99(7):891–900. doi: 10.1111/aogs.13811 (PMC7317858; doi:10.1111/aogs.13811)
Supplement: Supplementary file 5 [file AOGS-99-891-s005.docx]

Table 2. Model algorithms for prediction of gestational diabetes mellitus

| **Original study** | **The probability of developing gestational diabetes mellitus was calculated as e^lp^/(1+ e^lp^), where:** |
| --- | --- |
| Sweeting 2017 | Lp = -5.9515 + 2.8624 (if previous GDM) + 1.55 (if East Asian) + 1.7033 (if South Asian) + 3.0678 (if family history of DM) – 0.5836 (if multiparous) + 0.0354 (age, years) + 0.08462 (BMI, kg/m^2^). |
| Syngelaki 2015 | Previous GDM  Lp = -4.0050 + 3.9209 (if previous GDM) + 0.0206 (weight - 69, kg)  Nulliparous or parous with no previous GDM  Lp = -4.0050 – 0.7885 (if parous no previous GDM) + 0.0807 (age - 35, years) + 0.0381 (weight - 69, kg) – 0.0591 (height - 164, cm) + 0.9332 (if first degree relative with DM) + 0.5869 (if second degree relative with DM) + 0.4712 (if ovulation drugs) + 0.4562 (if Afro-Caribbean racial origin) + 1.0727 (if East Asian racial origin) + 0.8401 (if South Asian racial origin) + 0.2247 (birth weight z-score of previous pregnancy). |
| Eleftheriades 2014 | Lp = α + 0.058 (weight, kg) + 0.182 (age, years). |
| Gabbay-Benziv 2014 | Lp = -11.569 + 0.064 (age, years) + 2.026 (if Asian) + 0.083 (if black) + 0.493 (if Hispanic) + 1.661 (if other non-white ethnicity) + 2.144 (if prior GDM) + 0.034 (systolic blood pressure, mmHg) + 0.082 (BMI, kg/m^2^). |
| Tran 2013 | Lp = -8.22 + 0.11 (age, years) + 0.10 (BMI, kg/m^2^). |
| Syngelaki 2011 | Lp = α + 0.104 (BMI, kg/m^2^) + 0.068 (age, years) + 0.344 (if Afro-Caribbean) + 1.051 (if South Asian) + 1.278 (if East Asian) + 0.174 (if Mixed) + 0.432 (if ovulation drugs) + 0.315 (if IVF) + 0.020 (if smoker) – 0.010 (if history of chronic hypertension) – 0.211 (if parous without previous LGA infant) + 0.663 (if parous with previous LGA infant). |
| Teede 2011 | Lp = -5.31 + 0 (if age <25 years) + 0.92 (if age 25-29 years) + 1.22 (if age 30-34 years) + 1.69 (if age 35-39 years) + 1.95 (if age ≥40 years) + 0 (if BMI <20.0 kg/m^2^) + 0.53 (if BMI 20.0-24.9 kg/m^2^) + 0.69 (if BMI 25.0-26.9 kg/m^2^) + 0.83 (if BMI 27.0-29.9 kg/m^2^) + 1.28 (if BMI 30.0-34.9 kg/m^2^) + 1.82 (if BMI ≥35.0 kg/m^2^) + 0 (if Anglo-Australian) + 1.03 (if Polynesian) + 1.61 (if Mainland South-East Asian) + 1.13 (if Maritime South-East Asian) + 1.31 (if Chinese Asian) + 1.03 (if Southern Asian) + 0.69 (if African) + 0.18 (if other ethnicity) + 0.53 (if family history of DM) + 2.39 (if past history of GDM) + 0.26 (if poor obstetric outcome). |
| Nanda 2011 | Lp = -8.68947 + 0.05365 (age, years) + 0.10852 (BMI, kg/m^2^) + 1.00312 (if South Asian) + 0.88785 (if East Asian) + 3.72259 (if parous with previous GDM) + 0.67673 (if parous with previous LGA infant). |
| Van Leeuwen 2010 | Lp = -6.1 + 0.83 (if non-Caucasian ethnicity) + 0.57 (if positive family history of DM) – 0.67 (if multipara without history of GDM) + 0.5 (if multipara with history of GDM) + 0.13 (BMI, kg/m^2^). |
| Shirazian 2009 | Lp = α + 0 (if age ≤24 years) + 0.513 (if age 25-29 years) + 1.515 (if age ≥30 years) + 0 (if BMI ≤24.9 kg/m^2^) + 0.513 (if BMI 25.0-29.9 kg/m^2^) + 0.892 (if BMI ≥30.0 kg/m^2^) + 0.842 (if family history of DM type 2). |
| Phaloprakarn 2009 | Lp = α + 1.239 (age, years) + 2.187 (BMI, kg/m^2^) + 21.727 (if family history of DM) + 8.330 (if prior macrosomia) + 9.829 (if history of ≥2 abortions). |
| Naylor 1997 | Lp = α + 0 (if age ≤30 years) + 0 (if age 31-34 years) + 0.47 (if age ≥35 years) + 0 (if BMI ≤22 kg/m^2^) + 0.588 (if BMI 22.1-25.0 kg/m^2^) + 1.163 (if BMI ≥25.1 kg/m^2^) + 0 (if white ethnicity) – 0.357 (if black ethnicity) + 1.569 (if Asian) + 0.47 (if other ethnicity). |
| DM, diabetes mellitus; GDM, gestational diabetes mellitus; LGA, large-for-gestational-age; Lp, linear predictor | |
