# Supplementary material for: External validation and clinical utility of prognostic prediction models for gestational diabetes mellitus: A prospective cohort study
Source: Acta Obstet Gynecol Scand. 2020 Feb 14;99(7):891–900. doi: 10.1111/aogs.13811 (PMC7317858; doi:10.1111/aogs.13811)
Supplement: Supplementary file 6 [file AOGS-99-891-s006.docx]

**Table S3.** Characteristics included prediction models for external validation

| **First**  **Author (year)** | **Country** | **Study**  **design** | **Population** | **No. cases/total** | **Screening**  **GDM** | **Diagnosis**  **GDM** | **Definition**  **GDM** | **Statistical**  **model** | **Predictors** | **AUROC**  **(CI)** | **Internal validation**  **AUROC (CI)** | **External validation**  **AUROC (CI)** |
| --- | --- | --- | --- | --- | --- | --- | --- | --- | --- | --- | --- | --- |
| Sweeting (2017) | Australia | Case-control | 11^+0^ to 13^+6^ weeks GA  Exclusion: pre-existing DM, PE, multiple pregnancies, pre-term delivery (<37 weeks GA), miscarriage, stillbirth, termination, fetal chromosomal abnormality, missing clinical data and GDM diagnosed on a GCT alone. | 248/980 | Universal  All women 50g GCT at 24-28 weeks of gestation. Women at high risk for GDM were advised to undergo early OGTT; repeated at 18-20 weeks GA and 24-28 weeks GA if negative. | 2 hour  75-g OGTT | ADIPS  2014 | LR,  Akaike  information  criterion | Age  BMI  Ethnicity  Fam history DM  Parity  Prior GDM | 0.88  (0.85-0.92) | - | - |
| Syngelaki (2015) | United Kingdom | Prospective  cohort | 11^+0^ to 13^+6^ weeks GA  Exclusion: multiple pregnancy, pre-pregnancy DM type 1 or 2, and pregnancy ending in termination, miscarriage or delivery before 30 weeks GA, and major fetal defect. | 1827/75161 | Universal/Selective  Random plasma glucose at 24-28 weeks GA.  Persistent glucosuria or polyhydramnios, or if fetus becomes macrosomic. Other unit to women with risk factors. | 2 hour  75-g OGTT | WHO  1999 | LR,  backward | Age  Height  Weight  Ethnicity  Fam history DM  Method of conception  Parity  Prior GDM  Prior birth weight z-score | NR | 0.82  (0.82-0.83) | Australia:  0.87  (0.84-0.90)[^1^](#_ENREF_1) |
| Eleftheriades (2014) | Greece | Nested  case-control | 11-14 weeks GA  Exclusion, multiple pregnancy, hypertensive disorders of pregnancy, previous pregnancy complicated with GDM, pre-existing DM type 1 or 2, pregnancies with chromosomally abnormal foetuses and/or structural defects, pregnancies resulting in intra-uterine death or pregnancies diagnosed with severe early onset growth restriction. | 40/134 | Universal  2 hour 75-g OGTT at 24-28 weeks GA. | 2 hour  75-g OGTT | IADPSG  2010 | LR,  backward | Age  Weight | 0.73  (0.65-0.81) | - | Netherlands:  0.70  (0.65-0.74)[^2^](#_ENREF_2) |
| Gabbay-  Benziv (2014) | United States | Prospective  cohort | 11-14 weeks GA  Exclusion: multiple pregnancy, pre-GDM. | 63/924 | Universal  50-g GCT at 24-28 weeks GA. | 3 hour  100-g OGTT or single value >200 mg/dL on the GCT. | Carpenter  and Coustan  1982 | LR,  backward | Age  BMI  Ethnicity  Prior GDM  Systolic blood pressure | 0.82  (0.77-0.87) | - | Netherlands:  0.75  (0.71-0.79)[^2^](#_ENREF_2) |
| Tran (2013) | Vietnam | Prospective  cross-sectional  study | Routine antenatal care 24-32 weeks GA  Exclusion: ≤18 years old, multiple pregnancy, no delivery in inclusion hospital, and DM. | ADA 164/2772  ADIPS  577/2772  IADPSG  565/2772  WHO 674/2772 | Universal  2 hour 75-g OGTT at 24-28 weeks GA. | 2 hour  75-g OGTT | ADA  2010  ADIPS  1998  IADPSG  2010  WHO  1999 | LR | Age  BMI | ADA  0.71  (0.68-0.75)  ADIPS  0.64  (0.62-0.67)  IADPSG  0.65  (0.62-0.67)  WHO  0.63  (0.60-0.65) | - | Netherlands:  0.67  (0.63-0.72)[^2^](#_ENREF_2) |
| Syngelaki (2011) | United Kingdom | Prospective  cohort | 11^+0^ to 13^+6^ weeks GA  Exclusion: pre-pregnancy DM type 1 or 2, pregnancies conceived by intrauterine insemination, fetal aneuploidies or major defects, termination for psychosocial reasons, multiple pregnancy, and pregnancy ending in termination, miscarriage or delivery before 30 weeks of GA. | NR/41577 | Universal  Random plasma glucose at 24-28 weeks of gestation. | 2 hour  75-g OGTT | WHO  2006 | LR | Age  BMI  Ethnicity  Smoking  History of chronic hypertension  Method of conception  Parity  Prior LGA | NR | - | Netherlands:  0.71  (0.66-0.75)[^2^](#_ENREF_2) |
| Teede (2011) | Australia | Retrospective  cohort | 12 to 15 weeks GA  Exclusion: multiple pregnancy. | 250/2880 | Universal  75-g GCT at 26-28 weeks GA. | 2 hour  75-g OGTT | ADIPS  1998 | LR | Age  BMI  Ethnicity  Fam history DM  Prior GDM  History of poor obstetric outcome | NR | 0.70 | Netherlands:  0.77  (0.73-0.81)[^2^](#_ENREF_2)  Canada:  0.74  (0.70-0.78)[^3^](#_ENREF_3)  United Kingdom:  0.77  (0.76-0.77)[^4^](#_ENREF_4)  Italy:  0.60  (0.56-0.64)[^5^](#_ENREF_5) |
| Nanda (2011) | UK | Prospective  cohort | 11^+0^ to 13^+6^ weeks GA  Exclusion: multiple pregnancy, pre-pregnancy DM type 1 or 2, and pregnancy ending in termination, miscarriage or delivery before 30 weeks GA, and major fetal defect. | 297/11464 | Universal  Random plasma glucose at 24-28 weeks GA. Persistent glucosuria or polyhydramnios, or if fetus becomes macrosomic. | 2 hour  75-g OGTT | WHO  2006 | LR,  backward | Age  BMI  Ethnicity  Parity  Prior GDM  Prior LGA | 0.79  (0.76-0.82) | - | Netherlands:  0.78  (0.74-0.82)[^2^](#_ENREF_2)  United Kingdom:  0.79  (0.78-0.79) (overlap development cohort)[^4^](#_ENREF_4)  Australia:  0.78  (0.75-0.82)[^1^](#_ENREF_1) |
| Van Leeuwen (2010) | Netherlands | Prospective  cohort | <20 weeks GA  Exclusion: pregestational DM and multiple pregnancy. | 24/995 | Universal  Random glucose testing and 50-g GCT at 24-28 weeks GA. | 2 hour  75-g OGTT | WHO  1999 | LR,  backward | BMI  Ethnicity  Fam history DM  Parity  Prior GDM | 0.77  (0.69-0.85) | - | Netherlands:  0.74  (0.71-0.78)[^2^](#_ENREF_2)  Canada:  0.76  (0.73-0.79)[^3^](#_ENREF_3)  United Kingdom:  0.77  (0.77-0.78)[^4^](#_ENREF_4)  Australia:  0.64  (0.61-0.69)[^1^](#_ENREF_1) |
| Shirazian (2009) | Iran | Prospective  cohort | First visit  Exclusion: DM, presence of comorbid conditions, and GA >28 weeks at first visit. | 68/924 | Universal  2 hour 75-g OGTT at 24-28 weeks GA. | 2 hour  75-g OGTT | ADA  2007 | LR | Age  BMI  Fam history DM | NR | - | Netherlands:  0.71  (0.67-0.75)[^2^](#_ENREF_2) |
| Phaloprakarn (2009) | Thailand | Retrospective  cohort | ≤14 weeks GA  Exclusion: multiple pregnancy and DM. | 586/1876 | Universal  GCT at initial visit if risk factor and women without any risk factor at 24-28 weeks GA. High-risk women with a first normal GCT were retested at 28-32 weeks GA. | 3 hour  100-g OGTT | Carpenter  and Coustan  1982 | LR | Age  BMI  Fam history DM  Prior macrosomia  History of ≥2 abortions | 0.77  (0.75-0.79) | 0.75  (0.73-0.78) | - |
| Naylor (1997) | Canada | Prospective  cohort | <24 weeks GA  Exclusion: DM, <24 years of age, delivery <28 weeks GA, and multiple pregnancy. | 44/1560 | Universal  50-g GCT at 26 weeks GA and 3 hour 100-g OGTT at 28 weeks GA. | 3 hour  100-g OGTT | Carpenter  and Coustan  1982 | LR | Age  BMI  Ethnicity | 0.68 (NR) | NR | Netherlands:  0.64  (0.56-0.72)[^6^](#_ENREF_6)  0.72  (0.68-0.76)[^2^](#_ENREF_2)  Canada:  0.67  (0.64-0.70)[^3^](#_ENREF_3)  United Kingdom:  0.69  (0.68-0.69)[^4^](#_ENREF_4) |

ADA, American Diabetes Association; ADIPS, Australasian Diabetes in Pregnancy Society; AUROC, area under the receiving operating characteristic curve; BMI, body mass index; CI, confidence interval; DM, diabetes mellitus; GA, gestational age; GCT, glucose challenge test; GDM, gestational diabetes mellitus; IADPSG, International Association of the Diabetes and Pregnancy Study Groups; LGA, large-for-gestational-age; LR, logistic regression; NR, nor reported; OGTT, oral glucose tolerance test; PE, pre-eclampsia; WHO, World Health Organization

**References**

1. Sweeting AN, Appelblom H, Ross GP, Wong J, Kouru H, Williams PF, et al. First trimester prediction of gestational diabetes mellitus: A clinical model based on maternal demographic parameters. Diabetes Res Clin Pract. 2017;127:44-50.

2. Lamain-de Ruiter M, Kwee A, Naaktgeboren CA, de Groot I, Evers IM, Groenendaal F, et al. External validation of prognostic models to predict risk of gestational diabetes mellitus in one Dutch cohort: prospective multicentre cohort study. BMJ. 2016;354:i4338.

3. Theriault S, Forest JC, Masse J, Giguere Y. Validation of early risk-prediction models for gestational diabetes based on clinical characteristics. Diabetes Res Clin Pract. 2014;103:419-25.

4. Syngelaki A, Pastides A, Kotecha R, Wright A, Akolekar R, Nicolaides KH. First-Trimester Screening for Gestational Diabetes Mellitus Based on Maternal Characteristics and History. Fetal Diagn Ther. 2015;38:14-21.

5. Lovati E, Beneventi F, Simonetta M, Laneri M, Quarleri L, Scudeller L, et al. Gestational diabetes mellitus: including serum pregnancy-associated plasma protein-A testing in the clinical management of primiparous women? A case-control study. Diabetes Res Clin Pract. 2013;100:340-7.

6. van Leeuwen M, Opmeer BC, Zweers EJ, van Ballegooie E, ter Brugge HG, de Valk HW, et al. External validation of a clinical scoring system for the risk of gestational diabetes mellitus. Diabetes Res Clin Pract. 2009;85:96-101.
