# Supplementary material for: External validation and clinical utility of prognostic prediction models for gestational diabetes mellitus: A prospective cohort study
Source: Acta Obstet Gynecol Scand. 2020 Feb 14;99(7):891–900. doi: 10.1111/aogs.13811 (PMC7317858; doi:10.1111/aogs.13811)
Supplement: Supplementary file 7 [file AOGS-99-891-s007.docx]

Table S4. Characteristics of pregnancies in the observed and imputed validation cohort

| **Characteristics** | **Missing values,**  **n (%)**  **(n=5260)** | **Observed validation cohort**  **Complete cases^a^ (n=3925)** | **Observed**  **validation cohort**  **Women with missing value(s)^b^ (n=1335)** | **Imputed**  **validation**  **cohort**  **(n=5260)** |
| --- | --- | --- | --- | --- |
| Age (years) | 0 (0.0) | 30.4 (3.8) | 30.5 (3.9) | 30.4 (3.8) |
| Ethnicity, n (%)  - Caucasian  - Afro-Caribbean  - Indian, Pakistani or  Bangladeshi (South Asian)  - East Asian  - Other Asian  - Hispanic  - Mixed | 36 (0.7) | 3856 (98.2)  3 (0.1)  3 (0.1)  4 (0.1)  10 (0.3)  10 (0.3)  39 (1.0) | 1274 (95.4)  1 (0.1)  1 (0.1)  3 (0.2)  5 (0.4)  3 (0.2)  12 (97.3) | 5166 (98.2)  4 (0.1)  4 (0.1)  7 (0.1)  15 (0.3)  13 (0.2)  51 (1.0) |
| Tertiary education^c^, n (%) | 41 (0.8) | 2575 (65.6) | 854 (64.0) | 3445 (65.5) |
| Height (cm) | 20 (0.4) | 169.9 (6.4) | 170.3 (6.6) | 170.0 (6.5) |
| Weight (kg) | 24 (0.5) | 68.8 (12.4) | 68.6 (12.4) | 68.7 (12.4) |
| BMI^d^ (kg/m^2^) | 30 (0.6) | 23.8 (4.0) | 23.7 (4.1) | 23.8 (4.1) |
| Smoking during pregnancy, n (%) | 32 (0.6) | 152 (3.9) | 52 (3.9) | 205 (3.9) |
| History of chronic hypertension, n (%) | 17 (0.3) | 25 (0.6) | 5 (0.4) | 30 (0.6) |
| Family history of diabetes mellitus, n (%)  - First degree  - Second degree | 14 (0.3)  1 (0.0)  NM PRIDE Study | 512 (13.0)  747/2289 (32.6)  NM PRIDE Study | 158 (11.8)  108/313 (34.5)  NM PRIDE Study | 670 (12.7)  1671 (31.8) |
| Nulliparous, n (%) | 0 (0.0) | 2053 (52.3) | 711 (53.3) | 2764 (52.5) |
| Conception, n (%)  - Spontaneous  - Ovulation induction  - IVF/ICSI | 15 (0.3) | 3676 (93.7)  130 (3.3)  119 (3.0) | 1252 (93.8)  41 (3.1)  27 (2.0) | 4941 (93.9)  172 (3.3)  147 (2.8) |
| History recurrent miscarriages (≥2), n (%) | 0 (0.0) | 204 (5.2) | 71 (5.3) | 275 (5.2) |
| History of gestational diabetes mellitus, n (%) | 22 (0.4) | 21 (0.5) | 4 (0.3) | 25 (0.5) |
| History of macrosomia, n (%)  - >90^th^ percentile  - >95^th^ percentile  - ≥4000 g  - >4000 g | 96 (1.8)  96 (1.8)  103 (2.0)  103 (2.0) | 292 (7.4)  159 (4.1)  257 (6.5)  243 (6.2) | 92 (6.9)  47 (3.5)  87 (6.5)  84 (6.3) | 385 (7.3)  207 (3.9)  344 (6.5)  327 (6.2) |
| Birth weight z-score of previous pregnancy | 95 (1.8) | 0.2 (1.0) | 0.3 (1.0) | 0.2 (1.0) |
| History of poor obstetric outcome, n (%) | 67 (1.3) | 433 (11.0) | 166 (12.4) | 600 (11.4) |
| Systolic blood pressure (mmHg) | 1208 (23.0) | 114.3 (12.2) | 113.0 (11.8) | 114.4 (12.1) |
| Diastolic blood pressure^c^ (mmHg) | 1224 (23.3) | 67.5 (8.7) | 67.4 (9.3) | 67.6 (8.6) |
| ICSI, intracytoplasmic sperm injection; IVF, in vitro fertilisation; NM, not measured  ^a^All predictor values of the included models were complete  ^b^At least one missing value for a predictor of the included models  ^c^Not a predictor in the included models  ^d^Recoded/calculated on the basis of (imputed) original variables | | | | |
