# Supplementary material for: External validation and clinical utility of prognostic prediction models for gestational diabetes mellitus: A prospective cohort study
Source: Acta Obstet Gynecol Scand. 2020 Feb 14;99(7):891–900. doi: 10.1111/aogs.13811 (PMC7317858; doi:10.1111/aogs.13811)
Supplement: Supplementary file 8 [file AOGS-99-891-s008.docx]

Table S5. Baseline characteristics original cohorts and validation cohort (case-mix)

| **Characteristics** | **Sweeting**  **2017^a^** | | **Syngelaki**  **2015^a^** | | **Eleftheriades**  **2014^a^** | | **Gabbay-Benziv**  **2014** | | **Tran**  **2013** | **Syngelaki**  **2011^a^** | **Teede**  **2011** | **Imputed validation cohort** | | | | |
| --- | --- | --- | --- | --- | --- | --- | --- | --- | --- | --- | --- | --- | --- | --- | --- | --- |
|  | **GDM** | **No**  **GDM** | **GDM** | **No**  **GDM** | **GDM** | **No**  **GDM** | **GDM** | **No**  **GDM** |  |  |  | **All**  **n=5260** | **GDM**  **n=127** | | **No GDM**  **n=5133** | |
| Age (years)  - <25  - 25-29  - 30-34  - 35-39  - ≥40 | 33 | 32 | 33.1 | 30.7 | 33 | 30 | 32.7 | 29.8 | 28.2 (4.8) | 31.2 | 30.6 (5.5)  13.8%  29.6%  31.5%  19.7%  5.4% | 30.4 (3.8)  5.5%  36.3%  44.0%  13.3%  1.0% | 31.1 (4.1)  5.5%  32.3%  39.4%  22.0%  0.8% | | 30.4 (3.8)  5.5%  36.4%  44.1%  13.1%  1.0% | |
| Ethnicity (%)  - Caucasian  - Afro-Caribbean  - Asian  - Mainland South-East  - Maritime South-East  - Chinese  - Vietnamese  - Indian or Pakistani or  - Bangladeshi  - East  - Hispanic  - Anglo-Australian  - Polynesian  - Mixed/other | 43.5%  19.0%  36.7%  0.8% | 72.4%  7.4%  18.3%  1.9% | 59.8%  24.4%  9.2%  4.5%  2.1% | 75.1%  16.1%  4.2%  2.1%  2.4% | 100% | 100% | 33.3%  46.0%  15.9%  1.6%  3.2% | 46.8%  46.2%  3.9%  1.4%  1.6% | 95.3% | 75.5%  16.1%  4.1%  1.8%  2.5% | 4.7%  10.2%  4.1%  6.5%  11.8%  42.8%  1.7%  17.8% | 98.2%  0.1%  0.5%  0.1%  0.2%  0.1%  0.1%  0.1%  0.2%  1.0% | 97.6%  0.0%  2.4%  0.0%  0.8%  0.8%  0.8%  0.8%  0.0%  0.0% | | 98.2%  0.1%  0.4%  0.1%  0.1%  0.1%  0.1%  0.1%  0.3%  1.0% | |
| Tertiary education (%) |  |  |  |  |  |  |  |  | 28.2% |  |  | 65.5% | 51.2% | | 65.8% | |
| Height (cm) | 162.0 | 164.0 | 162.6 | 164.6 | 165.0 | 165.0 |  |  |  |  |  | 170.0  (6.5) | 168.8  (6.8) | | 170.0  (6.4) | |
| Weight (kg) | 64.4 | 63.7 | 76.7 | 66.0 | 69.0 | 63.0 | 86.9 | 74.2 |  |  |  | 68.7  (12.4) | 78.8  (16.2) | | 68.5  (12.2) | |
| BMI (kg/m^2^)  - <20.0  - 20.0-24.9  - 25.0-26.9  - 27.0- 29.9  - 30.0-34.9  - ≥35.0 | 24.5 | 23.3 | 29.0 | 24.3 |  |  | 32.6 | 27.6 | 20.6 (2.7) | 24.4 | 25.5 (6.6)  11.5%  39.2%  9.7%  9.2%  6.7%  8.0% | 23.8 (4.1)  14.3%  56.7%  11.1%  9.4%  6.8%  1.7% | 27.8 (6.0)  7.1%  35.4%  10.2%  9.4%  29.1%  8.7% | | 23.7 (3.9)  14.5%  57.2%  11.1%  9.4%  6.2%  1.5% | |
| Smoking during pregnancy (%) | 1.6% | 2.9% | 7.6% | 10.2% | 15.0% | 4.3% |  |  |  | 10.9% |  | 3.9% | 3.9% | | 3.9% | |
| History of chronic hypertension (%) |  |  |  |  |  |  | 11.1% | 6.3% |  | 1.1% |  | 0.6% | 0.0% | | 0.6% | |
| Family history of diabetes mellitus (%)  - First degree  - Second degree | 55.6% | 7.0% | 29.3%  10.3% | 13.0%  8.4% |  |  |  |  | 6.9% |  | 39.8% | 38.6%  12.7%  31.8% | 57.5%  30.7%  43.3% | | 38.1%  12.3%  31.5% | |
| Parity  Nulliparous (%) | 51.6% | 58.3% | 45.0% | 54.2% | 62.5% | 84.0% | 34.9% | 45.4% | 36.0% | NR |  | 0.6 (0.7)  52.5% | 0.6 (0.8)  55.9% | | 0.6 (0.7)  52.5% | |
| Conception (%)  - Spontaneous  - Ovulation induction  - IVF/ICSI | 94.8%  0.0%  5.2% | 95.9%  0.3%  3.8% | 94.8%  2.3%  2.9% | 96.7%  1.3%  2.0% |  |  |  |  |  | NR  NR  NR |  | 93.9%  3.3%  2.8% | 88.2%  7.1%  4.7% | | 94.1%  3.2%  2.7% | |
| History ≥2 miscarriages (%) |  |  |  |  |  |  |  |  |  |  |  | 5.2% | 6.3% | | 5.2% | |
| History of GDM (%) | 23.8% | 1.2% | 26.2% | 0.6% |  |  | 12.3% | 1.4% | 0.4% |  | 1.9% | 0.5% | 9.4% | | 0.3% | |
| History of macrosomia (%)  - >90^th^ percentile  - >95^th^ percentile  - ≥4000g  - >4000g | 6.5%^b^ | 0.0%^b^ |  |  |  |  |  |  | 1.1% | NR |  | 7.3%  3.9%  6.5%  6.2% | 16.5%  9.4%  11.8%  11.8% | | 7.1%  3.8%  6.4%  6.1% | |
| Prior birth weight z-score |  |  | 0.39  (1.3) | -0.04  (1.1) |  |  |  |  |  |  |  | 0.23  (1.0) | 0.84  (1.2) | | 0.22  (1.0) | |
| Poor obstetric outcome^b^ (%) |  |  |  |  |  |  |  |  |  |  | 3.6% | 11.4%  (Teede) | 13.4%  (Teede) | | 11.4%  (Teede) | |
| Systolic blood pressure (mmHg) |  |  |  |  |  |  | 124.1 | 115.1 |  |  |  | 114.4  (12.1) | 117.9  (12.4) | | 114.4  (12.1) | |
| Diastolic blood pressure (mmHg) |  |  |  |  |  |  | 70.8 | 67.5 |  |  |  | 67.6  (8.6) | 70.0  (8.9) | | 67.5  (8.6) | |
| GDM | 25.3% | | 2.4% | | 29.9% | | 6.8% | | ADA  5.9%  IADPSG  20.4%  WHO  24.3%  ADIPS  20.8% | NR | 8.9% | 2.4% | | | | |
| Predictors included in the model are shaded gray, continuous variables are presented as mean (SD)  ADA, American Diabetes Association; ADIPS, Australasian Diabetes in Pregnancy Society; GDM, gestational diabetes mellitus; IADPSG, International Association of the Diabetes and Pregnancy Study Groups; ICSI, intracytoplasmic sperm injection; IVF, in vitro fertilisation; NR, not reported  ^a^Continuous variables are presented as median  ^b^According to definition original article | | | | | | | | | | | | | |  | |  |

Table S5, continued. Baseline characteristics original cohorts and validation cohort (case-mix)

| **Characteristics** | **Nanda 2011^a^** | | **Van**  **Leeuwen**  **2010** | **Shirazian**  **2009** | **Phaloprakarn**  **2009** | | **Naylor**  **1997** | **Imputed validation cohort** | | |
| --- | --- | --- | --- | --- | --- | --- | --- | --- | --- | --- |
|  | **GDM** | **No**  **GDM** |  |  | **GDM** | **No**  **GDM** |  | **All**  **n=5260** | **GDM**  **n=127** | **No GDM**  **n=5133** |
| Age (years)  - <25  - 25-29  - 30-34  - 35-39  - ≥40 | 33.2 | 31.4 |  | 29.2%  40.3%  30.6% (>30) | 29.8 (5.8) | 25.6 (5.8) | 48.7% (≤30)  30.5% (31-34)  20.8% (≥35) | 30.4 (3.8)  5.5%  36.3%  44.0%  13.3%  1.0% | 31.1 (4.1)  5.5%  32.3%  39.4%  22.0%  0.8% | 30.4 (3.8)  5.5%  36.4%  44.1%  13.1%  1.0% |
| Ethnicity (%)  - Caucasian  - Afro-Caribbean  - Asian  - Mainland South-East  - Maritime South-East  - Chinese  - Vietnamese  - Indian or Pakistani or  - Bangladeshi  - East  - Hispanic  - Anglo-Australian  - Polynesian  - Mixed/other | 41.1%  43.8%  9.1%  3.4%  2.7% | 54.0%  35.6%  4.5%  2.2%  3.7% | NR |  |  |  | 81.5%  5.3%  9.0%  4.3% | 98.2%  0.1%  0.5%  0.1%  0.2%  0.1%  0.1%  0.1%  0.2%  1.0% | 97.6%  0.0%  2.4%  0.0%  0.8%  0.8%  0.8%  0.8%  0.0%  0.0% | 98.2%  0.1%  0.4%  0.1%  0.1%  0.1%  0.1%  0.1%  0.3%  1.0% |
| Tertiary education (%) |  |  |  | 25.3% |  |  |  | 65.5% | 51.2% | 65.8% |
| Height (cm) |  |  |  |  |  |  |  | 170.0  (6.5) | 168.8  (6.8) | 170.0 (6.4) |
| Weight (kg) |  |  |  |  |  |  |  | 68.7  (12.4) | 78.8  (16.2) | 68.5 (12.2) |
| BMI (kg/m^2^)  - <20.0  - 20.0-24.9  - 25.0-26.9  - 27.0- 29.9  - 30.0-34.9  - ≥35.0 | 29.4 | 24.2 | NR | 43.4% (≤24.9)  36.7% (25.0-29.9)  14.0% (≥30.0) | 24.4 (4.8) | 21.5 (3.5) | 52.8% (≤22.0)  26.6% (22.1-25.0)  20.6% (≥25.1) | 23.8 (4.1)  14.3%  56.7%  11.1%  9.4%  6.8%  1.7% | 27.8 (6.0)  7.1%  35.4%  10.2%  9.4%  29.1%  8.7% | 23.7 (3.9)  14.5%  57.2%  11.1%  9.4%  6.2%  1.5% |
| Smoking during pregnancy (%) | 6.7% | 7.7% |  |  |  |  |  | 3.9% | 3.9% | 3.9% |
| History of chronic hypertension (%) |  |  |  |  |  |  |  | 0.6% | 0.0% | 0.6% |
| Family history of diabetes mellitus (%)  - First degree  - Second degree | 23.9%  12.5% | 14.0%  13.4% | NR | 22.0% | 22.0% | 5.3% | 14.5% | 38.6%  12.7%  31.8% | 57.5%  30.7%  43.3% | 38.1%  12.3%  31.5% |
| Parity  Nulliparous (%) | 33.7% | 49.1% |  | 61.8% | 0.8 (0.8) | 0.6 (0.7) | 55.6% | 0.6 (0.7)  52.5% | 0.6 (0.8)  55.9% | 0.6 (0.7)  52.5% |
| Conception (%)  - Spontaneous  - Ovulation induction  - IVF/ICSI | 96.0%  4.0% | 97.4%  2.6% |  |  |  |  |  | 93.9%  3.3%  2.8% | 88.2%  7.1%  4.7% | 94.1%  3.2%  2.7% |
| History ≥2 miscarriages (%) |  |  |  | 2.8% | 3.1% | 0.5% |  | 5.2% | 6.3% | 5.2% |
| History of GDM (%) | 21.2% | 0.4% | NR |  |  |  |  | 0.5% | 9.4% | 0.3% |
| History of macrosomia (%)  - >90^th^ percentile  - >95^th^ percentile  - ≥4000g  - >4000g | 17.8% | 5.0% |  |  | 3.8% | 0.5% |  | 7.3%  3.9%  6.5%  6.2% | 16.5%  9.4%  11.8%  11.8% | 7.1%  3.8%  6.4%  6.1% |
| Prior birth weight z-score |  |  |  |  |  |  |  | 0.23  (1.0) | 0.84  (1.2) | 0.22  (1.0) |
| Poor obstetric outcome^b^ (%) |  |  |  |  |  |  | 12.8% | 11.4%  (Teede) | 13.4%  (Teede) | 11.4%  (Teede) |
| Systolic blood pressure (mmHg) |  |  |  |  |  |  |  | 114.4  (12.1) | 117.9  (12.4) | 114.4  (12.1) |
| Diastolic blood pressure (mmHg) |  |  |  |  |  |  |  | 67.6  (8.6) | 70.0  (8.9) | 67.5  (8.6) |
| GDM | 2.6% | | 2.4% | 7.4% | 31.2% | | 2.8% | 2.4% | | |
| Predictors included in the model are shaded gray, continuous variables are presented as mean (SD)  ADA, American Diabetes Association; ADIPS, Australasian Diabetes in Pregnancy Society; GDM, gestational diabetes mellitus; IADPSG, International Association of the Diabetes and Pregnancy Study Groups; ICSI, intracytoplasmic sperm injection; IVF, in vitro fertilisation; NR, not reported  ^a^Continuous variables are presented as median  ^b^According to definition original article | | | | | | | | | | |
