# Supplementary material for: External validation and clinical utility of prognostic prediction models for gestational diabetes mellitus: A prospective cohort study
Source: Acta Obstet Gynecol Scand. 2020 Feb 14;99(7):891–900. doi: 10.1111/aogs.13811 (PMC7317858; doi:10.1111/aogs.13811)
Supplement: Supplementary file 9 [file AOGS-99-891-s009.docx]

**File S1 – Excluded studies**

*Algorithm not available*

1. Lu L, Koulman A, Petry CJ, Jenkins B, Matthews L, Hughes IA, et al. An Unbiased Lipidomics Approach Identifies Early Second Trimester Lipids Predictive of Maternal Glycemic Traits and Gestational Diabetes Mellitus. Diabetes Care. 2016; 39:2232-2239.

2. Theriault S, Giguere Y, Masse J, Girouard J, Forest JC. Early prediction of gestational diabetes: a practical model combining clinical and biochemical markers. Clin Chem Lab Med. 2016; 54:509-518.

3. Savvidou M, Nelson SM, Makgoba M, Messow CM, Sattar N, Nicolaides K. First-trimester prediction of gestational diabetes mellitus: examining the potential of combining maternal characteristics and laboratory measures. Diabetes. 2010; 59:3017-3022.

*Model already published in one of the included articles*

4. Syngelaki A, Visser GH, Krithinakis K, Wright A, Nicolaides KH. First trimester screening for gestational diabetes mellitus by maternal factors and markers of inflammation. Metabolism. 2016; 65:131-137.

5. Hassiakos D, Eleftheriades M, Papastefanou I, Lambrinoudaki I, Kappou D, Lavranos D, et al. Increased Maternal Serum Interleukin-6 Concentrations at 11 to 14 Weeks of Gestation in Low Risk Pregnancies Complicated with Gestational Diabetes Mellitus: Development of a Prediction Model. Horm Metab Res. 2016; 48:35-41.

6. Papastefanou I, Eleftheriades M, Kappou D, Lambrinoudaki I, Lavranos D, Pervanidou P, et al. Maternal serum osteocalcin at 11-14 weeks of gestation in gestational diabetes mellitus. Eur J Clin Invest. 2015; 45:1025-1031.

7. Ferreira AF, Rezende JC, Vaikousi E, Akolekar R, Nicolaides KH. Maternal serum visfatin at 11-13 weeks of gestation in gestational diabetes mellitus. Clin Chem. 2011; 57:609-613.
